# Validation of a modified QuEChERS method for the quantification of residues of currently used pesticides in Cuban agricultural soils, using gas chromatography tandem mass spectrometry

**DOI:** 10.1007/s11356-024-33237-6

**Published:** 2024-04-30

**Authors:** Brizeidi Peña, Dayana Sosa, Isabel Hilber, Arturo Escobar, Thomas Daniel Bucheli

**Affiliations:** 1 Analytical Unit of Residues and Contaminants, National Center for Animal and Plant Health (CENSA), San José de las Lajas, P.O. Box 10, 32700 Mayabeque, Cuba; 2 Agroscope Environmental Analytics, Reckenholzstrasse 191, 8046 Zurich, Switzerland

**Keywords:** Pesticides, Tropical soils, Matrix effects, QuEChERS, Quality assurance and control

## Abstract

We present an analytical method to detect and quantify residues of currently used pesticides (CUPs), which include 31 active ingredients (ai) and seven transformation products (TPs) in tropical and agricultural soils of Cuba. Ten isotopically labeled analogous compounds served as internal standards (IL-IS). The novelty of this research is the inclusion of different tropical soils type scarcely studied for CUPs and TPs, based on the QuEChERS (quick, easy, cheap, effective, rugged and safe) method, followed by chromatography tandem mass spectrometry. All figures of merit proved to be satisfactory according to SANTE guidelines 2020 and 2021. Matrix effects (ME) calculated by the external standard method were significant (|ME| > 20% for almost all compounds; grand mean ± standard deviation (*STD*) 104 ± 108%) in all soils. The internal standard method compensated ME to non-significant levels (8 ± 50%), even for analytes with a non-structure identical IL-IS (STD, 13 ± 57%). Repeatability (relative standard deviation, *RSD*_*r*_) and reproducibility (*RSD*_*R*_) for skeletic regosol (*SR*) were 7.5 ± 2.8% and 11.7 ± 4.7%, respectively. Absolute (quantified for 11 analytes with structure identical IL-IS) and relative recovery from SR was 92 ± 13% (*mean* ± *STD*) and 90 ± 12%, respectively. Limits of quantification for *SR* ranged from 0.1 to 10 ng/g, except metalaxyl and oxyfluorfen (25 ng/g each). Linearity of matrix-matched (MM) calibration curves (5 to 100 ng/g) had an *R*^2^ of ≥ 0.99 for all soils and almost all analytes. The method was successfully applied to 30 real soil samples.

**Supplementary Information:**

The online version contains supplementary material available at 10.1007/s11356-024-33237-6.

## Introduction

Conventional agriculture has been applying pesticides to control pests such as weeds, insects, and fungi and maintain high levels of productivity (Hvězdová et al. 2018, Li 2022). According to the Food and Agricultural Organization of the United Nations (FAO), the global agricultural use of pesticides in 2020 was 2.7 Mt of active ingredients (ai), and the average application per crop area was 1.8 kg/ha (FAO 2022). The Americas applied the highest level of pesticides in agriculture with an average of more than 1 Mt pesticides/year and also topped the levels of pesticides per cropland with 2.8 kg/(ha × year) during 1990–2020 (FAO 2022).

Despite the benefits of pesticides in terms of food production and crop yields (Silva et al. 2019), their intensive and long-term application can negatively impact the environment (Sabzevari  and Hofman 2022, Wang et al. 2020). Currently used pesticides (CUPs) are considered more environmental friendly, effective and safe than for instance the banned organochlorine pesticides (Hvězdová et al. 2018). However, some CUPs can still persist in soil (Geissen et al. 2021, Riedo et al. 2021), appear in remote areas (Hvězdová et al. 2018), contaminate plant tissues (Kalyabina et al. 2021), affect food safety (Carvalho 2017, Seneff 2021, Zikankuba et al. 2019), occur in drinking or surface waters (de Souza et al. 2020, Syafrudin et al. 2021), and generally represent a risk for human health (Lewis et al. 2016, Seneff 2021, Sidhu et al. 2019), and the environment (Carvalho 2017, Lewis et al. 2016, Md Meftaul et al. 2020, Tudi et al. 2021, Zikankuba et al. 2019).

Once the pesticides are applied, agricultural soils are among the first exposed environmental media. Recently, a number of articles of residues of CUPs in agricultural soils of temperate regions such as Europe have been published (Hvězdová et al. 2018, Padilla‐Sánchez et al. 2015, Riedo et al. 2023, Sabzevari and Hofman 2022, Silva et al. 2019). However, in tropical areas, monitoring of CUPs is limited (Tan et al. 2020), and research was mainly conducted in Asian countries (Sabzevari and Hofman 2022), first of all China (Tan et al. 2020) and India (Kumari et al. 2008, Murugan et al. 2013). In tropical areas of Latin America and the Caribbean, only a few reports exist of Brazil (Bortolozo et al. 2016, Guarda et al. 2020), Mexico García-Hernández et al. 2021, Velasco et al. 2012), and Argentina (Primost et al. 2017).

Although the access to pesticides in Cuba is limited due to the trade and financial embargos, crops such as potato, tomato, and beans having a high national demand are prioritized when it comes to the distribution of CUPs, which happens on a national level. The most recent monitoring of pesticide residues in Cuba dates back to 1996, where concentrations ranged from 90 (desmetryn, a triazine) to 1960 μg/kg (diuron, a phenylurea) in soil 60 to 164 days after harvest (Dierksmeier 1996). Limits of detection of the 11 compounds analyzed in soil ranged from 20 to 300 μg/kg. Dierksmeier (1996) already used multi-residue techniques almost 30 years ago, but emphasized the need for more sensitive methods especially when it comes to food samples whose regulated maximum residue levels (MRL) need to be analytically met. The author (Dierksmeier 1996) concluded, after having monitored Cuban soil, water, and some cash crops such as beans, potato and tomato, that the island needs more monitoring studies in zones of agricultural impact.

Therefore, the aim of this paper was to develop and validate a multi-residue method for CUPs in agricultural, tropical soils, or different types. The method is based on QuEChERS (quick, easy, cheap, effective, rugged and safe) extraction (Anastassiades et al. 2003a), compounds are measured by gas chromatography coupled to tandem mass spectrometry (GC-MS/MS) and quantified with internal standard method. The target analyte list includes ai approved and mostly used in Cuban contemporary agriculture and some of their transformation products (TPs) with a focus on potato being a crop to which most pesticides are applied in Cuba. Whenever available and applicable in MS/MS, isotope-labeled analogues were used as internal standard (IL-IS). The method should be easy to transfer to Cuban laboratories and similar equipment such as GC-single quadrupole MS, GC-electron capture detector (ECD), and GC-nitrogen phosphorus detection, that are more available in developing and tropical countries. It was applied to a set of 30 soil samples of 150 from a 4-year-monitoring campaign between 2018 and 2022, with the purpose to quantify CUP residues in different agricultural soils of potato production. The results of this campaign will serve to derive regulatory guidance values (RGVs) for Cuban soils (Jennings and Li 2014).

## Material and methods

### Reagents and standards

Table S1 of the supporting information (SI) lists all pesticides, TPs, and isotope-labeled internal standards (IL-IS) used in this study, their CAS numbers, providers and purities ranging from 93 to 100%. Most of the compounds were dissolved in acetonitrile (ACN), except prosulfocarb and dimethomorph that were in ethanol (EtOH), to obtain stock solutions and stored at −20 °C. All solvents used (HiPersolv Chromanorm for HPLC-ultra LC-MS) were purchased from VWR Chemicals BDH (Dietikon, Switzerland). Primary secondary amine sorbent (PSA, 40 μm, Bondesil) was purchased from Supelco, Bellafonte, USA. Magnesium sulfate (MgSO_4_, anhydrous reagent Plus ≥ 99.5%), dried in a muffle oven at 500 °C for at least 4 h, and sodium acetate (CH_3_COONa, puriss p.a. ACs reagent, and anhydrous) were purchased from Sigma-Aldrich (Merck & Cie, Schaffhausen, Switzerland), sodium chloride (NaCl, for analysis) from Merck (Darmstadt, Germany), and formic acid (FA, Analar NORMAPUR, 99-100%) from VWR Chemicals BDH (Dietikon, Switzerland).

### Selection of pesticides

The compound list (Tables S1 and S2) contained 38 ai of which 39% were fungicides (F), 24% herbicides (H), and 18% insecticides/acaricides (I/A). Additionally, some TPs (18%) as referred to in the database of pesticide properties (PPDB 2023) and based on availability on the market were added to the list (Tables S1 and S2). The CUPs were selected according to the official list of pesticide applications to potato crop in Cuba (MINAG 2016, 2022) but also comprised legacy compounds such as endosulfanes (α and β) and their TP (endosulfane sulphate). They were included because, although banned in Cuba since 2013 (Pérez-Consuegra and Montano-Pérez 2021), they continued in use (MINAG 2016) until 2022 (MINAG 2022). Prosulfocarb and carfentrazone-ethyl have not been approved for use in Cuba at least for 10 years (MINAG 2008) but were studied as negative control because they have frequently been applied in temperate agroecosystems (EC 2019, Hvězdová et al. 2018). Table S2 shows some physicochemical properties of all analytes.

### Soils, sampling, and sample preparation

The soil type rhodic ferralic nitisol (RFN, classification according to World Reference Base for soil (WRB 2015)) is predominant in the Mayabeque province of Cuba and appropriate for agriculture (Febles-González et al. 2013). However, an agricultural soil may contain ai and/or TPs that limit its use for method development and validation (i.e. quantification by a matrix-matched (MM) calibration and as a negative, blank control). Thus, other soils possibly without residues were additionally needed. Table 1 lists the different soil types (skeletic regosol (SR), RFN, xanthic ferralic nitisol (XFN), and dystric cambisol (DC)) taken for MM calibration to preliminarily quantify pesticide residues and to study effects of the matrix. Rhodic ferralic nitisol was included twice, i.e., as organically and conventionally managed, respectively, because soil parameters were not the same in the two of them. The SR, a forest soil, was used for the validation because it originates from a natural park where pesticides were never applied.

**Table 1:** Physico-chemical characteristics of the soils used for method development and validation

Soil type according World Reference Base (WRB 2015)	Skeletic regosol	Rhodic ferralic nitisol		Xanthic ferralic nitisol	Dystric cambisol
Abbreviation	SR	RFNo	RFNc	XFN	DC
Use	Forestry	Agriculture organically managed	Agriculture conventionally managed	Agroforestry for animal feed	Grass
Texture of the soil	Clay loam	Silty loam	Clay loam	Silty clay	Silty clay
pH	6.1	7.3	6.8	7.5	8.0
Organic matter (%)	3.1	2.6	1.7	3.5	1.9
Silt (%)	26.0	50.5	40.4	40.6	41.6
Sand (%)	34.5	27.9	26.7	13.9	10.7
Clay (%)	34.2	19.0	30.3	40.6	44.8

The soils were sampled in the Mayabeque province, Cuba (Fig. S1) between 2014 and 2021 (Table S3) according to the Swiss soil monitoring network NABO, which is described in Gubler et al. (2015). In a 10 m × 10 m area 100 soil cores were taken with an auger of 1.8-cm diameter at a soil depth of 20 cm. Twenty-five of the soil samples were bulked to one sub-sample, immediately brought to the lab, dried in an air-conditioned room of 25 °C at 5% humidity for 7 days until constant weight, crushed, and sieved over a 2-mm sieve and stored in the dark until analysis.

### Preparation of target analytes, IL-IS, syringe standard, and calibration solutions

Stock solutions of each ai or TP around 2000 µg/mL were dissolved in ACN (or EtOH, see “Reagents and standards”). A working mixture standard solution was diluted by mixing the stock solutions of each compound with ACN to a concentration of 1 µg/mL. The 10 IL-IS (Table S1) were individually dissolved in ACN obtaining stock solutions with a concentration around 1000 µg/mL. Then, an IL-IS mixture standard solution was produced in ACN at 0.5 µg/mL. Triphenyl phosphate (TPP) was used as syringe standard. It was dissolved in ACN at a concentration of 2000 µg/mL and diluted in ACN at 0.5 µg/mL. All solutions were prepared gravimetrically.

Analyte solutions for calibration (0, 5, 10, 25, 50, 75, and 100 ng/mL) were prepared from dilutions of the working standard mixture in ACN with FA 2.5% (ACN/FA). The IL-IS mixture and TPP (Table S1) were added in each calibration point at a concentration of 10 ng/mL each. All solutions such as stock, intermediate, and the working solutions were stored at −20 °C in the dark until use.

### Extraction and clean-up by the QuEChERS method

The methods of Acosta-Dacal et al. (2021) and Rösch et al. (2023) were applied with modifications. Briefly, soil prepared as described in “Soils, sampling, and sample preparation” was mixed vigorously for 5 min in a Turbula shaker-mixer (Willy A. Bachofen AG, Muttenz, Switzerland) before taking a subsample of 5 g. It was put into a centrifuge tube (15 mL), and 10 ng/g IL-IS mixture was added (0.1 mL IL-IS solution). After 30 min of rest to allow the ACN to evaporate, the sample was rehydrated with 5 mL of Milli-Q water (Valverde et al. 2021), vortexed for 1 min, and kept at room temperature for 1 h. Next, 5 mL ACN/FA was added to the sample in the centrifuge tube, vortexed for 3 min, and sonicated for 15 min at 35 kHz with 80% microprocessor control (Sonorex Digital 10 P, Bandelin from IG, Zurich, Switzerland). Then, 2 g of MgSO_4_, 0.5 g NaCl, and 0.5 g CH_3_COONa were added to the mixture (Mahdavi et al. 2021), vortexed immediately for 1 min, homogenized with a Turbula for 10 min, and sonicated for 15 min with the same settings as indicated above. Subsequently, the sample was centrifuged for 10 min at 4200 rpm in a 5804 R Eppendorf centrifuge (Eppendorf, Hamburg, Germany). One mL of supernatant extract was filtered through a 0.20-μm Chromafil® PET filter (Macherey-Nagel, Düren, Germany). Finally, 0.02 mL of TPP corresponding to 10 ng/g was added as syringe standard before injection. An aliquot of 0.5 µL of the extract (see below) was directly injected into GC-MS/MS. The pesticide extraction with the procedure described before was compared to one with an additional clean-up step (0.05 g PSA, 2 g MgSO_4_, and 0.5 g CH_3_COONa). No differences in the pesticide concentrations were observed applying these extraction methods. However, we choose to apply the additional clean-up step for protecting the GC capillary column and the MS under routine analysis.

### Instrument conditions

#### GC-MS/MS system

A GC-MS/MS (GC-2030 system MS-TQ8050 NX model, Shimadzu Corp., Kyoto, Japan) with an AOC-20i+s Plus (auto-injector and auto-sampler coupled to the GC) and an electronic flow controller were used in this study. Instrument control, data acquisition, and processing were performed using the Shimadzu GCMSolution software (version 4.45). Data were quantified by the LabSolutions Insight (version 3.1).

The analytes were separated on an SH-Rxi-5ms capillary column (30 m × 0.25 mm id and 0.25-μm film thickness; Shimadzu, USA). Helium 5.0 (> 99.999% purity, PanGas AG, Dagmersellen, Switzerland) was used as carrier gas (head pressure was set at 92 kPa with total flow at 26.6 mL/min, column flow at 1.35 mL/min, linear velocity at 46.2 cm/s, and purge flow at 5 mL/min). The injector temperature was at 280 °C (Shimadzu recommendation: 200–300 °C, Shimadzu GCMS-TQ series Jul 2018, 225-38195) and injection took place at high pressure of 250 kPa for 1 min onto a Topaz Liner with glass wool, split 3.5 mm × 5.0 × 95 for Shimadzu GCs (Cat #23319 from Restek, BGB Analytik, Boeckten, Switzerland). The oven temperature gradient program was as follows: hold an initial temperature of 80 °C (1.5 min, Shimadzu recommendation: 40–100 °C, Shimadzu GCMS-TQ series Jul 2018, 225-38195), ramp to 300 °C with 25 °C/min, and hold for 11 min. The total run time was 21.3 min. The solvent delay was 4 min; the retention time of the compounds is between 6 and 14 min (Table S4); and the column is reconditioned at 300 °C for 8 min.

The MS analyses were conducted in positive electron ionization (EI+) mode with a filament current set at 60 μA, ionization energy at 70 eV, and detector voltage at 1.9 kV. The ion source and transfer line temperatures were heated at 300 °C each. For ion fragmentation, argon 5.0 (> 99.999% purity, PanGas AG, Dagmersellen, Switzerland) was used as collision-induced dissociation gas. The optimization of chromatographic and mass spectrometric conditions from injection until finding the optimal transitions of the analytes is explained in the SI (“Optimization of chromatographic and mass conditions”).

#### Quantification of pesticides and transformation products

Quantification was done with the internal standard method (normalization of analyte peak area against one of the corresponding IL-IS) with a MM calibration and concentrations of 0, 5, 10, 25, 50, 75, and 100 ng/mL. All calibration regressions were calculated with the linear least square method and NOT forced through the origin. Ideally, each compound would have a corresponding structure identical (si) IL-IS, which was not possible. The parent ions of deuterated IL-IS were often the same as for native compounds due to the strong ionization in the source of the GC-MS/MS. Hence, only 10 IL-IS had different parent ions than their native analytes and could be added to the compound list in Table S4 for identification and quantification of compounds with si IL-IS. Please note that the IL-IS n-methyl-metribuzin-D3 was used for both, metribuzin desamino (DA), and desamino diketo (DADK) as si IL-IS (Table S4, IL-IS group 4). For this reason, there are 10 IL-IS but 11 analytes with si IL-IS. All other compounds (27) are quantified with a non-structural (nsi) IL-IS, where the IL-IS group is given in Table S1 and the corresponding analyte in Table S4.

### Method validation

The validation of the method was primarily preformed according to the newest SANTE guideline for soil matrices (SANTE 2020). For reproducibility, the SANTE guideline for food and feed (SANTE 2021) was used. While individual validation parameters are additionally evaluated for all of the soils listed in Table 1, the complete validation is carried out for SR due to the reasons outlined in “Soils, sampling, and sample preparation.” Analytical parameters evaluated were ME, slopes of MM calibration curves of the different soils, repeatability and reproducibility (as measures of precision), absolute and relative recovery (the latter as a measure of trueness (SANTE 2021)), limit of quantification (*LOQ*), linearity, selectivity, and specificity. Each of them is briefly described in “Matrix effect” to “Selectivity and specificity” and “Statistics and data presentation,” and corresponding results presented and discussed in chapters “Matrix effects of different soils, matrix matched calibrations and the role of isotope-labeled internal standards,” “Precision (repeatability (*RSDr*) and reproducibility (*RSDR*)) and trueness for skeletic regosol (*SR*),” “Limit of quantification (*LOQ*) and linearity for SR,” and “Selectivity and specificity for SR.”

#### Matrix effect

While a comparison between internal and external standards (without normalization of analyte peak area against one of the corresponding IL-IS) method in soil extracts was carried out for all compounds, and more specifically for compounds with si IL-IS (Hartmann et al. 2007), to show ME, the routine analysis exclusively used the former. The concentrations of the standards in the ACN/FA solutions were the same as in MM calibrations.

The influence of co-extracts on the chromatographic signal, i.e., the ME, was evaluated by comparing the slopes of the calibration curves used for external (ext) standard method (Eq. (1a)), and internal (int) standard method (Eq. (1b)), respectively, of the individual compounds in MM calibration from the five soils (Table 1) with those in ACN/FA. The ME was quantified with Eqs. (1a) and (1b) and considered significant if |ME| > 20% according to the SANTE guidelines (SANTE 2020). A suppression is an ME smaller than −20% and an enhancement larger than 20% (SANTE 2020).

1a $${{\text{ME}}}_{{\text{ext}}} \left(\mathrm{\%}\right)=\left(\frac{{\mathrm{slope\;of\;MM\;calibration}}_{\mathrm{ext }} }{{\mathrm{slope\;of\;calibration\;in\;solvent}}_{{\text{ext}}} }-1\right)\times 100$$

1b $${{\text{ME}}}_{{\text{int}}}\left(\mathrm{\%}\right)=\left(\frac{{\mathrm{slope\;of\;MM\;calibration}}_{\mathrm{int }} }{{\mathrm{slope\;of\;calibration\;in\;solvent}}_{{\text{int}}} }-1\right)\times 100$$

#### Precision and trueness as a measure of accuracy

Precision and trueness are two complementary sides of accuracy, the closeness of an analytical result to a true value, where the former covers random and the latter systematic analytical errors (SANTE 2021). Precision was calculated as relative standard deviation (*RSD*) of quadruplicate analyses at each of three concentration levels (10, 25, and 50 ng/g), analyzed at the same day, and at three different days, to yield repeatability (*RSD*_*r*_), and reproducibility (*RSD*_*R*_), respectively (Table 2). The highest concentration (50 ng/g) corresponds to the acceptable *LOQ* for pesticide residue analyses in soil (EC 2010). Reproducibility was only included in the SANTE guideline (SANTE 2021) for food and feed with ≤ 20%. The Horwitz function describes the relation of *RSD*_*r*_ and *RSD*_*R*_ (originally: coefficients of variation) of multiple analyses as a function of analyte concentration, which serves here as an additional evaluation criterion (Horwitz 1982).

A significant ME (“Matrix effect”) may lead to low or high analyte recoveries, which can, however be accounted for by a MM calibration (Anastassiades et al. 2003b, Łozowicka et al. 2017). To reduce the variability of the recoveries (absolute and relative) induced by different soil types in a set of samples, MM calibrations were applied. Absolute recoveries of the 10 IL-IS determined from four replicates at the three fortification levels were quantified by dividing the ratio of the area of IL-IS (nominal concentration 10 ng/g, ISO-13876, 2013) (ISO 2013) spiked before extraction over the area of TPP (nominal concentration 10 ng/g, ISO-13876, 2013) (ISO 2013) spiked before injection over the ratio of area_IL-IS_/area_TPP_ from the MM calibration. The IL-IS/TPP spiking time shift compensated for losses of the correspondent native compounds during preparation and clean-up and for ME. In contrast, relative recovery determined for all target analytes is the relation of analyte concentration quantified with MM calibration over the one spiked at a given concentration into the soil prior to extraction (Table 2). As such, it is a measure of trueness.

#### LOQ and linearity

The LOQ was the lowest detectable concentration of the MM calibration (0.1, 0.25, 0.5, 1, 5, 10, 25, 50, 75, and 100 ng/mL), with a signal of the quantifier ion transition over the noise ratio (*S*/*N*) >10 and the *S*/*N* ratio of the qualifier ion transition > 3. MM calibration curves were checked for linearity in the concentration levels (5, 10, 25, 50, 75, and 100 ng/ml). The 0 ng/mL was not included in the calibration curve because the data acquisition program did not allow a “non”-concentration. The range of analyzed concentrations was within the range of *LOQ* to 500 × *LOQ*. The calibration was carried out by the IL-IS method (IL-IS concentration: 10 ng/mL). The linearity was expressed as coefficient of determination *R*^2^ and *RSD*.

#### Selectivity and specificity

Selectivity is the unique identification of an analyte by its retention time, which was the middle of the base of the peak ± 0.5 min, and the quantifier to qualifier ion transition ratio, given by the respective MM calibration ± 50% for a sample. Specificity is the differentiation of the analyte from components in the matrix or other compounds of the method. This parameter is especially important when working, as in our case, with deuterated IL-IS and the corresponding native compound in mass spectrometer techniques where the ionization is harsh and *m*/*z* of their fragments often the same. Abundance and concentration of each pesticide of (i) the lowest MM calibration level (5 ng/g), (ii) SR blanks, and (iii) SR fortified at the lowest level for each matrix (10 ng/g) were compared qualitatively (Fig. S4). The chromatograms should show a clear peak/resolution especially between the 5 and 10 ng/g concentrations and no peaks in the SR blank, and the latter should, according to the SANTE (2020) guidance, not be higher than 30% of the *LOQ*. Otherwise, detailed justification has to be provided. Both parameters were not only part of the validation but contribute to quality control and quality assurance (QA/QC) under routine analysis.

### Method application to real Cuban soil samples

The validated method was applied to 30 samples of Cuban soils taken from 14 different potato fields (Fig. S1; Table S3). The samples represent a subset of a large monitoring campaign with different pesticide use of conventional (*n* = 26) and organic (*n* = 4) farming of RFN soil type. Sampling and preparation of soil samples were carried out as described in “Soils, sampling, and sample preparation.” Two extraction series were conducted, consisting each of 15 samples, three spiked RFNo (10, 25, 50 ng/g, *n* = 1 for each concentration), non-spiked RFNo in triplicates (*n* = 6 in total), three non-spiked sand samples (*n* = 6 in total), and 12 non-spiked SR (blank SR from the reproducibility batch of 3 days in quadruplicates/day). All non-spiked samples served as blanks. Reproducibility as a measured of extended method precision in RFNo was assessed by the corresponding coefficient of variation (*RSD*) of relative recoveries for each spiked level (10, 25, and 50 ng/g). Ten ng/g of IL-IS was spiked before extraction, and TPP were added before injection to each sample according to “Extraction and clean-up by the QuEChERS method.” All concentrations were calculated using a MM calibration curve with RFNo from the organic potato field as its soil was essentially pesticide-free as well, but this was only known after the method had been developed. The results are discussed in “Application of the method to real samples.”

### Statistics and data presentation

**Fig 1:**
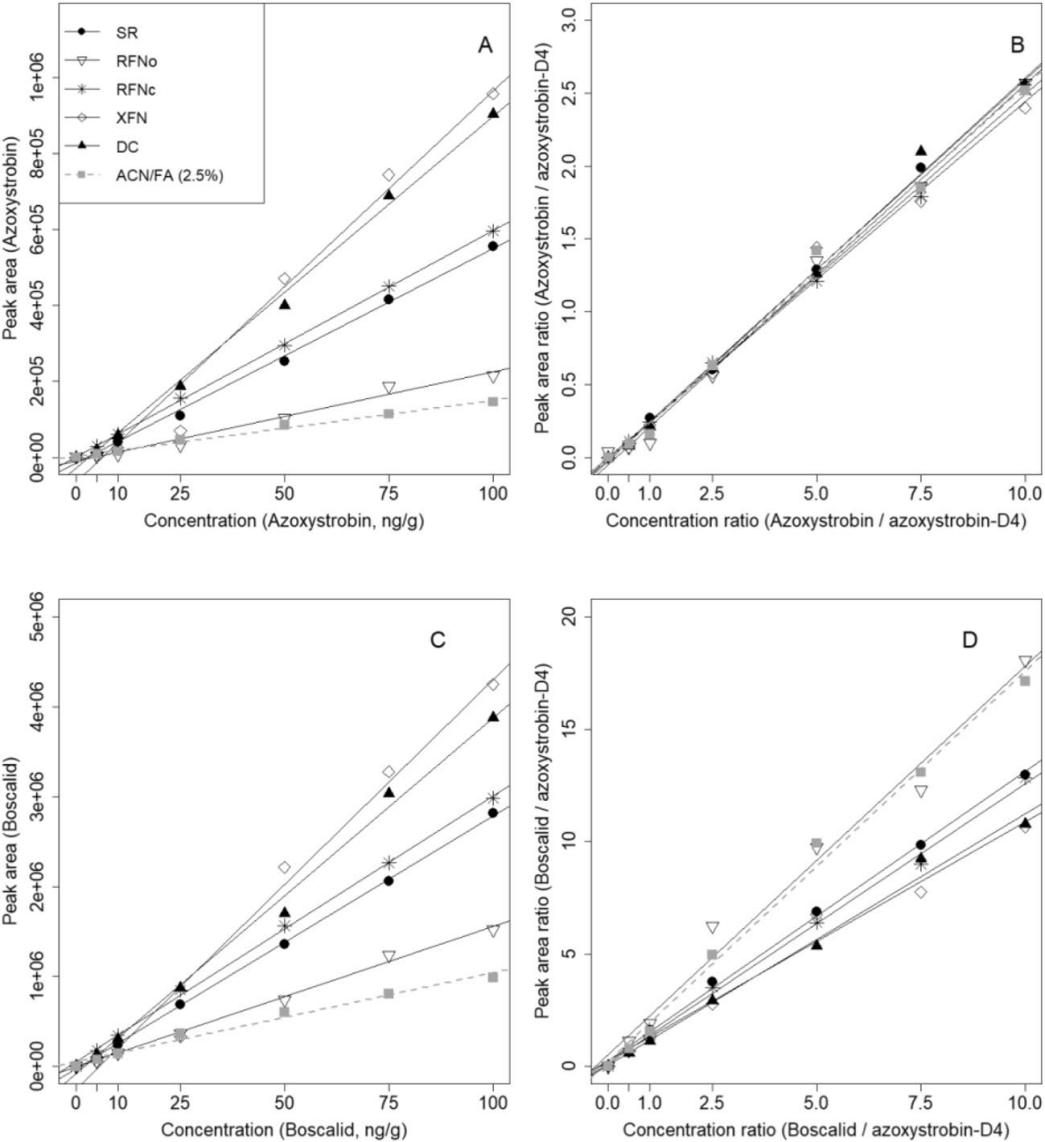
Matrix-matched external (panels **A** and **C**) and internal (panels **B** and **D**) calibration curves of azoxystrobin (panels **A** and **B**) and boscalid (panels **C** and **D**). Azoxystrobin-D4 was used as a structure identical isotope labeled internal standard (IL-IS) for azoxystrobin (panel B), and as a non-structure identical IL-IS for boscalid (panel D). Matrix-matched calibrations are from different soil types: skeletic regosol (SR, black dots), rhodic ferralic nitisol, organically managed (RFNo, transparent triangle), and conventionally managed (RFNc, star), xantic ferralic nitisol (XFN, transparent diamond), and dystric cambisol (DC, black triangle). The solvent was acetonitrile with 2.5% (v/v) formic acid (ACN/FA, gray box).

**Fig 2:**
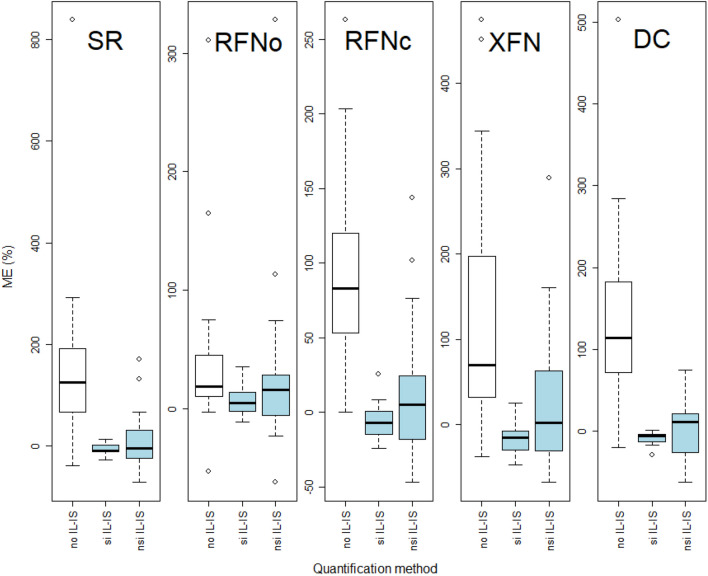
Matrix effect (ME) of skeletic regosol (SR), rhodic ferralic nitisol organically managed (RFNo), RFN conventionally managed (RFNc) xanthic ferralic nitisol (XFN), and dystric cambisol (DC). Please note that the *y*-axes have different scales. White boxes represent ME of all 38 analytes quantified with external standard calibrations without isotope labeled internal standards (IL-IS) according to Eq. (1a), and blue ones ME determined with the internal standard method and IL-IS with Eq. (1b). Eleven compounds had a structure identical isotopically labeled internal standard (si IL-IS), and 27 compounds were quantified with non-structure identical (nsi) IL-IS. The boxes represent the 25^th^ to the 75^th^ percentiles; the whiskers are the 10^th^ and 90^th^ percentiles; the dots are outliers; and horizontal, black bars in the boxes are medians.

**Fig 3:**
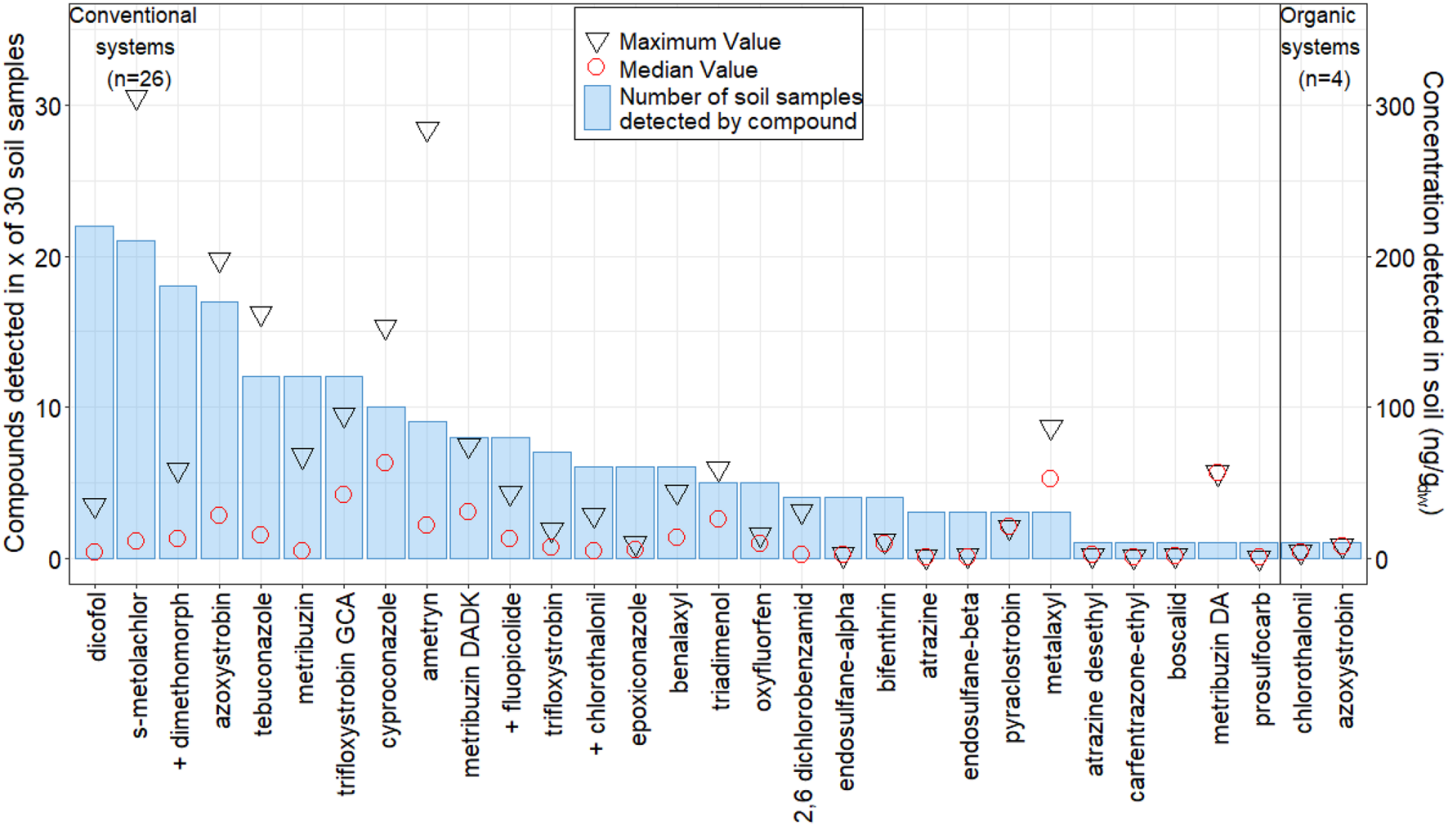
Pesticide residues quantified in 30 Cuban soil samples. Blue bars (left *y*-axis) indicate how many times a respective compound was detected. The right *y*-axis reports median (empty red circles) and maximum (empty black triangles) concentrations (ng/gdw) in the soil samples. From the top-ten most applied pesticides to potato crops according to Barroso Planas (2014) compounds that coincided with the investigated ai are marked with a “+” in *x*-axis labels.

Descriptive statistics (Figs. 1, 2, and 3) and statistical data evaluation were carried out with R version 4.0.2 (2020-06-22). The slopes of the calibration curves of all compounds of SR, the soil used for the validation of the method, were compared against all other soils (Table 1) with the null hypothesis H_0_ that the slope *β*_1_ = *β*_2_ or *β*_1_ − *β*_2_ = 0 to evaluate its suitability. A Student’s *t*-test according to Eq. (2) described in Andrade and Estévez-Pérez (2014) assuming unequal variances was used for comparison.

2 $$t= \frac{{b}_{1}- {b}_{2}}{\sqrt{{{s}^{2}}_{b1}+ {{s}^{2}}_{b2}}} \sim T\left({n}_{1} {+ n}_{2}\right)$$

where *b* is the slope of the MM calibration of the different soils, and *s*^2^_*b*1_, *s*^2^_*b*2_ are the variances of the respective slopes of the first order linear regressions. In most cases of compound calibrations, sample size was equal (*n*_1_ = *n*_2_), and *t*-test results were the same whether variances were pooled or not (Andrade and Estévez-Pérez 2014).

## Results and discussions

### Method validation

#### Matrix effects of different soils, matrix-matched calibrations, and the role of isotope-labeled internal standards

In GC-MS, along the way from the injector to the detector, different processes occur that enhance or suppress the signals of the target analytes, leading to a ME. On the one hand, the presence of matrix components protects the analytes from adsorption or degradation during evaporation in the inlet (Acosta-Dacal et al. 2021, Asensio-Ramos et al. 2010). On the other hand, both the chromatographic separation and signal-to-noise ratios of target analytes, as well as their ionization efficiency in the ion source might be disturbed by overwhelming matrix constituents.

The ME manifests directly and pronouncedly in the comparison of matrix-matched external calibrations. Figure 1 A and C present exemplary calibration curves of azoxystrobin and boscalid, respectively, in a pure solvent (ACN/FA) and in extracts of different soil types. In all cases, the slopes of the latter exceeded those of the former, by up to a factor of four. Such ME can effectively be compensated for by normalizing peak areas (and respective concentrations) to those of a suitable IL-IS, as illustrated in Fig. 1 B for azoxystrobin and its si IL-IS azoxystrobin-D4. No si IL-IS was available for boscalid, but normalizing it to azoxystrobin-D4 as a nsi IL-IS (Fig. 1D) reduced slope variabilities of different soil types by roughly a factor of two. Slopes and corresponding ME of both external and internal calibrations of all analytes are listed in Tables S5 and S6, respectively.

Applying Eq. (1a) of the *external standard method* for all soil types leads to the corresponding ME_ext_. The overall mean of the ME_ext_ of 38 compounds over the five soils was 104% and the standard deviation (STD) ±108%. Hence, the majority of the soils exhibited a significant ion enhancement (ME_ext_ > 20%, Fig. 2, white boxes). While the RFNo had the lowest mean ME_ext_ over all compounds with 34%, the other soils had mean ME_ext_ ranging from 89% (RFNc) to 146% (SR, Table S5). First and third quartiles of the ME_ext_ ranged from 11% in RFNo to 190% in SR (Fig. 2, Table S5). Hence, half of the values (1^st^ to 3^rd^ quartiles) span from 57% (RFNo) to as much as 254% (DC, please note the different *y*-axis scales in Fig. 2). Ion enhancement occurred in 45 to 97% of the 38 compounds in all soil types and went as high as 839% (Fig. 2), with dicofol exhibiting maximum ME_ext_ in all soils (ME_ext_ from 263 to 839%, Table S5). Ion suppression (ME < −20%) of all soils and compounds was not as relevant as enhancement except for pyraclostrobin representing the minimum ME_ext_ or the highest ion suppression in all soil types. The RFNo had the lowest number of significant ME_ext_ (16 out of 38 analytes) and SR and DC had all analytes with a significant ME_ext_ (Table S5). The ME_ext_ of individual analytes in the different soils was compared with their respective properties (Table 1), in particular their OM content, but no correlation with any of them could be observed. This result is unlike the one for another method recently developed in our lab, also with different soils but measured with liquid chromatography MS/MS, where the ME increased with increasing OM and concomitantly shifted towards ion suppression (Rösch et al. 2023).

Secondly, ME_int_ by Eq. (1b) was calculated using the *internal standard method*, which entails the normalization of analyte peak areas against the ones of IL-IS. As expected, with a mean of 8% and a *STD* ± 50% for all 38 compounds over the five soils, the ME_int_ were drastically smaller than the ME_ext_. Similarly, the median ME_int_ of compounds with si IL-IS (*n* = 11) were closer to 0% than those of analytes with nsi IL-IS (*n* = 27) (Fig. 2, blue boxes, Table S6). However, as ME_int_ are normalized values, they do not discriminate between suppression or enhancement anymore. Means of ME_int_ of compounds with si IL-IS ranged from -17% (XFN) to 7% (RFNo) and laid more in the negative than in the positive but within the non-significant span demanded by SANTE guideline (SANTE 2020). The means of ME_int_ of compounds with nsi IL-IS span from 3 to 27%, again meeting the SANTE requirements, except for RFNo with 27%. First and third quartiles of the ME_int_ of compounds with si IL-IS ranged from −30% in XFN to 14% in RFNo (Fig. 2, Table S6). Hence, the span (difference) of the 1^st^ to the 3^rd^ quartiles of the ME_int_ for compounds with si IL-IS of SR, RFNo/c, and DC was as narrow as 14 to 17%, except for XFN that was 37%. Although the 1^st^ to the 3^rd^ quartiles of the ME_int_ for compounds with nsi IL-IS was broader (−31 to 63%, both in XFN) than for the ones with si IL-IS, the behavior was the same because the span of ME_int_ was 34 to 54% for SR, RFNo/c, and DC with the exception of XFN (94%). Of course, the measurement by GC-MS/MS (“Quantification of pesticides and transformation products”) invoked the different number of compounds in the IL-IS groups, 11 versus 27, which obviously also made the variation of the ME_int_ smaller in the former than the latter group. However, the comparison of ME_int_ of nsi IL-IS with the ME_ext_ (Fig. 2; no IL-IS) revealed the advantage of using IL-IS, even if nsi: the ME_ext_ were factors bigger than those of analytes with nsi IL-IS, except for RFNo (Fig. 2). Hence, the use of IL-IS, irrespective of si or nsi, did not only compensate the ME more than 10 times in comparison to the external standard method (see above) but also its variability, which is in line with that of Homazava et al. (2014).

The assumption for single analytes was again that the ME_int_ for compounds with si IL-IS would be smaller than for those with nsi IL-IS. Indeed, no significant (according to SANTE) ME_int_ were observed for 2,6-dichlorobenzamid, metribuzin desamino (DA), metalaxyl, fluopicolide, epoxiconazole, and azoxystrobin in all five soil types. These compounds, except epoxiconazole, had an si IL-IS. In contrast and as expected, compounds with nsi IL-IS as prosulfocarb and oxyfluorfen showed a significant ME_int_ in all five soils (Table S6) and fluazifop-p-butyl, trifloxystrobin CGA, benalaxyl, endosulfane sulfate, tebuconazole, bifenthrin, fenamidone RPA, and pyraclostrobin in four soil types. Pyraclostrobin showed significant ME_ext_ and ME_int_ in all soils of the former and four soils of the latter method. Between 12 and 20 compounds per soil type had no significant |ME_int_| (< 20%) (Table S6) which is according to what other authors reported (Bortolozo et al. 2016; Łozowicka et al. 2017).

The differences of slopes in calibration curves evoked above by different soil matrices could be substantially reduced by IL-IS, be it si or nsi (Fig. 1). However, the slopes of the MM calibration curves calculated with the internal standard method of the different soils still needed to be compared to know whether SR, the soil validated here was suitable, especially as RFNo, is the one used in routine. This was statistically expressed in “Statistics and data presentation” by the H_0_ that the slope *β*_1_ = *β*_2_ or *β*_1_ − *β*_2_ = 0 of an analyte in soil_1_ and soil_2_, where the desired outcome would be H_0_ *not rejected* and calculated according to Eq. (2). The slopes of compounds with a si IL-IS agreed in more cases, meaning they showed no significant difference in the *t*-test, than compounds with nsi IL-IS. Specifically, SR versus RFNo slopes showed in 82% of compounds with si IL-IS (*n* = 11) and no significant difference, and in 30% with nsi IL-IS (*n* = 27), versus RFNc slopes 64% and 37%, versus DC 82% and 56%, and versus XFN 18% and 11%, respectively. Slopes of SR with XFN correlate the least, which is also seen in Fig. S5. The smallest correlation coefficient *r* for the other soils was with the solvent (ACN_FA), which again underlines the necessity for MM calibrations. Furthermore, 64% of compounds with si IL-IS show no significant differences of slopes when RFNo is compared to RFNc and 33% of nsi IL-IS. This is also reflected in the correlation coefficient *r* = 0.89 in Fig. S5 when RFNo and RFNc are correlated. Hence, even the same soil type but managed differently exhibited significantly different slopes, which might be due to the different soil properties of the RFN (Table 1). This slope comparison including the aforementioned findings emphasize the use of as many as possible IL-IS even for analytes with nsi IL-IS when dealing with different soils and relativized the question of SR’s suitability.

#### Precision (repeatability (*RSD*_*r*_) and reproducibility (*RSD*_*R*_) and trueness for skeletic regosol (SR)

Table 2 lists accuracy expressed as precision and trueness obtained with the here presented method. The means of repeatability (*RSD*_*r*_) for the 10, 25, and 50 ng/g fortification levels were 9.2, 7.3, and 5.5%, respectively, with a clear tendency of higher precision with higher fortification concentration. These results met well the quality criterion of the SANTE guideline (SANTE 2020) of *RSD*_*r*_ ≤ 20%. Only metribuzin DADK and bifenthrin slightly exceeded 20% *RSD*_*r*_ at 10 ng/g (20.4 and 20.8%), while their *RSD*_*r*_ of 25 and 50 ng/g complied with the quality criterion (SANTE 2020). The *RSD*_*r*_ at different fortification levels were lowest for 2,6-dichlorobenzamid (1.6, 2.5, and 2.8 ng/g) and highest for bifenthrin (20.8, 15.3, and 9.3 ng/g). The precision in terms of reproducibility (*RSD*_*R*_) is generally lower, i.e., has a higher percentage than *RSD*_*r*_ because it is obtained under changing conditions (SANTE 2021), in this case at different days. Indeed, the *RSD*_*R*_ means of all compounds and fortification levels were higher than for *RSD*_*r*_ and were 15.5, 11.8, and 7.3% again with increasing precision for increasing concentration. Minima ranged from 3.1% (metribuzin DADK at 50 ng/g) to 4.3% (2,6-dichlorobenzamid at 25 ng/g), and maxima from 15.4% (dicofol at 50 ng/g) to 36.6% (bifenthrin at 10 ng/g). The SANTE guideline for food and feed (SANTE 2021) sets the within-laboratory reproducibility (*RSD*_*wR*_, which is the same as our *RSD*_*R*_) at ≤ 20%. All of the values met this criterion at the highest fortification level, 34 out of 38 compounds at 25 ng/g and 30 out of 38 at the lowest level. This is a satisfactory performance for concentrations at the ppb level. Horwitz considers the within-laboratory *RSD* (*RSD*_*wl*_), which is corresponding to our *RSD*_*R*_, to be two-thirds of the between laboratories (*RSD*_*bl*_ (%) = 2^(1−0.5×logC)^, where *C* is the concentration) and thus around 30% for organic compounds (Horwitz 1982). So, also according to Horwitz, the *RSD*_*R*_ of this study were good with only three compounds at the lowest fortification level (10 ng/g) exhibiting an *RSD*_*R*_ >30%: endosulfane sulfate (35.1%), bifenthrin (36.6%) and pyraclostrobin (30.9%). Such precision ranges also compare well with those of earlier papers in this domain (Łozowicka et al. 2017, Rösch et al. 2023).

**Table 2:** Method validation figures of merit with relative standard deviation (*RSD*) of repeatability (*RSD*_*r*_) and reproducibility (*RSD*_*R*_) as measure of precision, relative recovery as measure of trueness, limit of quantification (*LOQ*), and linearity of matrix-matched calibration curves in skeletic regosol. Means were derived from quadruplicates of each fortification level and validation parameter. Compounds, type of pesticides, and the corresponding isotopically labeled internal standards (IL-IS) group are shown in columns 2–4 from the left.

No.	Analyte	Type^a^	IL-IS group^b^	*RSD*_*r*_ (%) (repeatability)			*RSD*_*R*_ (%) (reproducibility)			Relative recovery (%)			*LOQ* (ng/g)	Linearity (*R*^2^)
				Fortification level (ng/g)										
				**10**	**25**	**50**	**10**	**25**	**50**	**10**	**25**	**50**		
1	Metribuzin DADK	TP	4	20.4	6.9	2.2	28.2	11.1	3.1	50.2	82.6	97.3	10	0.9976
2	Atrazine desethyl	TP	1	8.4	2.6	2.9	16.9	9.7	4.2	99.1	87.7	93.1	1	0.9983
3	2,6-Dichlorobenzamid	TP	2	1.6	2.5	2.8	4.9	4.3	3.7	108.1	115.4	107.7	5	0.9955
4	Atrazine	H	1	4.7	12.6	4.5	7.4	13.3	5.8	99.9	100.7	95.0	1	0.9958
5	Clomazone	H	1	2.7	2.5	4.6	4.7	7.9	5.9	80.6	80.1	79.1	0.5	0.9989
6	Chlorothalonil	F	4	4.3	4.2	6.2	7.8	9.9	8.0	92.8	121.2	114.8	0.1	0.9972
7	Metribuzin DA	TP	4	11.1	4.2	3.3	16.9	7.5	4.3	80.9	89.6	92.2	10	0.9906
8	Pirimicarb	I/A	5	5.4	2.5	5.3	20.9	13.8	7.3	77.6	84.3	81.6	5	0.9991
9	Metribuzin	H	5	4.3	4.0	3.6	8.3	6.2	4.7	88.2	97.3	97.3	1	0.9988
10	Ametryn	H	5	8.0	5.0	2.5	11.6	12.1	6.1	74.8	78.7	78.1	10	0.9988
11	Metalaxyl	F	6	11.5	4.8	5.0	15.0	8.1	6.5	108.5	95.9	103.3	25	0.9975
12	Prosulfocarb	H	6	18.6	5.7	7.8	24.6	9.5	10.1	84.3	95.3	103.0	0.25	0.998
13	S-metolachlor	H	7	7.1	7.1	5.6	13.3	9.5	7.4	106.9	104.6	98.8	5	0.9958
14	Dicofol	I/A	7	14.1	13.1	11.8	27.5	20.3	15.4	65.2	74.8	78.5	1	0.9965
15	Trifloxystrobin CGA	TP	8	5.6	9.7	5.8	8.9	13.2	7.5	73.8	86.0	77.0	5	0.9963
16	Triadimenol	F	7	2.6	4.2	2.8	9.5	5.7	3.7	92.2	94.8	93.0	5	0.9993
17	α-Endosulfane	I/A	7	7.7	7.2	6.0	10.5	11.7	7.8	74.0	81.0	83.6	0.1	0.9976
18	Oxyfluorfen	H	7	9.5	6.8	4.9	12.6	9.2	6.3	97.3	97.4	97.2	25	0.993
19	Fluazifop-p-butyl	H	7	8.1	4.6	3.6	14.4	7.4	4.8	106.0	101.7	101.5	0.1	0.9997
20	Cyproconazole	F	8	2.7	6.3	5.4	3.8	8.4	7.0	82.2	73.2	70.5	5	0.9987
21	β-Endosulfane	I/A	7	10.4	6.5	7.1	18.4	10.9	9.2	92.3	91.9	92.9	10	0.9952
22	Carfentrazone-ethyl	H	8	7.1	6.5	4.0	9.5	8.7	5.2	89.1	89.3	91.5	1	0.995
23	Trifloxystrobin	F	8	6.0	6.3	5.3	7.7	8.2	6.8	88.9	81.6	79.4	5	0.9988
24	Benalaxyl	F	8	9.7	6.3	6.1	13.1	8.5	7.8	109.2	83.1	82.2	5	0.9982
25	Fluopicolide	F	9	11.6	5.1	5.9	15.7	7.2	7.6	112.3	106.9	104.4	10	0.9969
26	Endosulfane sulphate	TP	9	8.9	9.0	8.9	35.1	27.8	12.2	97.0	97.0	98.8	0.1	0.9933
27	Tebuconazole	F	9	12.2	10.2	7.5	16.4	13.5	9.7	108.9	111.2	111.0	0.5	0.9989
28	Epoxiconazole	F	9	9.5	8.6	6.1	14.6	14.8	7.9	83.4	92.9	89.4	0.1	0.9961
29	Bifenthrin	I/A	9	20.8	15.3	9.3	36.6	21.8	12.4	66.9	60.9	57.7	10	0.9962
30	Fenamidone	F	9	7.5	9.1	5.0	14.8	15.3	6.6	93.6	86.9	84.2	5	0.9967
31	Benthiavalicarb-isopropyl	F	9	7.7	7.9	5.6	13.9	13.7	7.3	89.3	93.0	89.5	1	0.9977
32	Fenamidone RPA	TP	9	14.6	11.6	7.3	22.3	17.4	9.6	95.9	106.9	106.1	0.25	0.9972
33	Pyraclostrobin	F	9	15.2	13.1	6.0	30.9	21.0	8.2	112.2	78.2	65.6	10	0.9985
34	Spirotetramat	I/A	9	12.3	11.9	5.0	16.5	17.3	6.5	80.6	82.5	79.6	5	0.9937
35	Boscalid	F	10	9.7	8.3	6.0	13.6	11.3	7.8	86.7	88.7	83.6	0.1	0.9987
36	Deltamethrin	I/A	10	12.1	11.6	9.0	17.9	15.1	11.6	78.0	79.8	75.8	10	0.9995
37	Azoxystrobin	F	10	8.0	4.8	3.9	13.1	7.0	5.1	101.8	100.7	99.1	0.5	0.9979
38	Dimethomorph	F	10	6.5	7.1	4.7	10.0	9.8	6.0	87.7	90.4	87.7	0.1	0.9975
	Mean			9.2	7.3	5.5	15.5	11.8	7.3	89.9	91.2	90.0	5	
	Min			1.6	2.5	2.2	3.8	4.3	3.1	50.2	60.9	57.7	0.1	
	Max			20.8	15.3	11.8	36.6	27.8	15.4	112.3	121.2	114.8	25	

^a^Type of pesticide: *I*/*A* insecticide/acaricide, *F* fungicide, *H* herbicide, *TP* transformation product

^b^IL-IS group refers to the 10 IL-IS in Table S4. As there were fewer IL-IS than compounds, several analytes were assigned to one IL-IS. As an example, prosulfocarb was quantified with the IL-IS metalaxyl-D6, as was metalaxyl

Absolute recoveries of IL-IS spiked to SR were 92% ± 20% (*mean* ± *STD* of the means of three fortification levels in Table S7). Although the SANTE guideline (SANTE 2020) sets the limit for relative recoveries, we consider 70–120% for absolute recoveries as satisfactory too. Exceptions were n-methyl-metribuzin-D3 with an absolute recovery of 43% ± 4% and azoxystrobin-D4 of 123 ± 23% (Table S7). Absolute recoveries from RFNo under routine method execution are presented in “QA/QC in RFNo soils under routine operation.” The application of a larger number of IL-IS, be it si or nsi, and reporting of their absolute recoveries represents an advantage over other multi-residue methods that only used a few internal standards or IL-IS. For instance, Vu-Duc et al. (2023) established a method for 21 organochlorinated compounds by GC-MS/MS in different soil samples (*n* = 6) using α-HCH-D6 as internal standard, but absolute recovery was not evaluated. Acosta-Dacal et al. (2021) presented a method for 51 pesticides by GC-MS/MS, and only two IL-IS were used. However, the paper does not report any result of absolute recoveries to compare our results with.

For relative recoveries, representing accuracy in terms of trueness, 100% corresponds to the “true” value. The means of relative recovery from fortified samples in SR ranged from 89.9 to 91.2% (min = 50.2%, max = 121.2%, Table 2). The SANTE guideline (SANTE 2020) proposes relative recoveries between 70 and 120% for matrices other than food/feed of plant and animal origin. Hence, the relative recoveries were satisfactory. Almost all analytes met the criterion, except bifenthrin that presented a mean relative recovery of 62% and was similarly low in all three fortified levels (67, 61, and 58%, Table 2). This might be due to its high octanol-water partition coefficient (log K_OW_) of 6.6 (Table S2) indicating strong sorption to, and/or incomplete extraction from the soil’s organic carbon. Metribuzin DADK (50% at 10 ng/g), chlorothalonil (121% at 25 ng/g), dicofol (65% at 10 ng/g), and pyraclostrobin (65% at 50 ng/g) showed relative recoveries outside the acceptable range for individual spike levels. However, their overall mean recovery of these analytes was still in the range of 70–120%.

These results are similar to the ones other authors reported. In contrast to the SANTE guideline (SANTE 2020), some researchers considered the method for routine analysis in GC-MS/MS fit for purpose when compounds had a mean relative recovery between 60 and 140% if the compound had a good precision (Acosta-Dacal et al. 2021, Łozowicka et al. 2017). For instance, Łozowicka et al. (2017) employed the QuEChERS method without cleanup for 216 pesticides and metabolites, analyzed the extract with GC-MS/MS, obtained a recovery in a range of 71–120% for all pesticides, except for five of them (recovery between 65 and 69%) and considered this result satisfactory. Acosta-Dacal et al. (2021) found recoveries in the range of 70–120% for 59% of the analytes quantified by QuEChERS and GC-MS/MS (51 compounds in total) for all fortification levels ranging from the highest concentration (50 ng/g) to the *LOQ* set for each analyte. However, 18 of them exhibited recoveries over 120% (121–131%), and three had recoveries below 70% (60–69%). These compounds were nevertheless included in the method for routine analysis.

In summary, precision (*RSD*_*r*_ ≤ 20% and *RSDR* ≤ 30%) and relative recoveries (60–140%) (Acosta-Dacal et al. 2021, Łozowicka et al. 2017, Horwitz 1982) were satisfactory, for mostly compounds. Some minor exceedances for relative recoveries were recorded for metribuzin DADK, chlorothalonil, and dicofol but are nevertheless trustful due to the good precision. If relative recovery and precision for a compound were low (i.e. bifenthrin and pyraclostrobin), concentrations between *LOQ* and lowest spiking limit (10 ng/g) need to be interpreted with care for SR. Therefore, soil type specific QA/QC should be included when applying method for soil monitoring.

#### LOQ and linearity for SR

The *LOQs* of the different pesticides spiked to SR ranged from 0.1 to 25 ng/g with a mean of 5 ng/g (Table 2). All values were in the same order of magnitude as those of other soil studies using tandem MS coupled to GC (Acosta-Dacal et al. 2021, Łozowicka et al. 2017, Słowik-Borowiec et al. 2022). All compounds had an *LOQ* between 2 and 500 times lower than required by the SANTE guideline (50 ng/g) (SANTE 2020), except metribuzin DADK showing a peak in the unspiked SR (red line of first panel in Fig. S4) close to *LOQ* (10 ng/g, Table 2), and are thus fit-for-purpose as a pesticide soil exposure assessment tool. Linearity data (equation and *R*^2^) of calibration curves were from concentrations ranging from 5 to 100 ng/mL in the SR matrix obtained in triplicates using the internal standard method (Table 2). Coefficient of determination (*R*^2^) were satisfactory (i.e., > 0.99) for all analytes.

#### Selectivity and specificity for SR

The lowest fortification level (10 ng/mL) in SR was compared with the lowest calibration point in solvent ACN/FA (5 ng/mL) and with an uncontaminated SR soil (blank, Fig. S4). Transitions of qualifier ions were depicted too (Fig. S3). Each analyte could be detected without interference from impurities (< 30% *LOQ*), degradation products or excipients present in the matrix except metribuzin DADK (“LOQ and linearity for SR”).

### Application of the method to real samples

#### QA/QC in RFNo soils under routine operation

The above outlined method was routinely applied to a subset of Cuban soils, and the results are presented here. Real samples were mainly from RFN soil with the majority RFNc, but QA/QC was performed with RFNo due to reasons outlined in Soils, sampling, and sample preparation. Absolute recoveries from RFNo (IL-IS mix spiked before extraction, TPP spiked before injection) were satisfactory (overall mean ± *STD* = 102% ± 10%; derived from of the means of two extractions series) for all IL-IS (Table S8) and in the same range as those of SR (see above), but with a smaller STD. Relative recoveries of most analytes in RFNo laid within the satisfactory range of 60 to 140%, with good reproducibility (*RSD*_*R*_ < 30%) (Table S9). The lowest spiked level (10 ng/g) had the highest number of compounds with recoveries < 60% or > 140% (metribuzin DADK, trifloxystrobin CGA, carfentrazone-ethyl, benalaxyl, fenamidone, fenamidone RPA, and spirotetramat). Spirotetramat showed a low recovery (< 60%) and high reproducibility value (*RSD*_*R*_ > 30%) at 10 ng/g. Concentration of compounds with recoveries (< 60% or > 140%) and *RSD*_*R*_ (> 30%) out of range need to be interpreted carefully (Table S9).The SR showed slightly less compounds outside the desired range (Table 2), which is a better performance than RFNo. The *LOQ* of RFNo ranged from 0.1 to 25 ng/g, which were the same min and max as for SR, but the former soil had a slightly lower mean (Table S9 and Table 2). Different blank samples in RFNo showed signals below *LOQ*, except for metribuzin DADK. Similar to SR (“LOQ and linearity for SR” and “Selectivity and specificity for SR”), Sand and RFNo blanks run under routine revealed metribuzin DADK clearly above the *LOQ* of 5 ng/g for RFNo (Table S10). Sources of cross contamination were searched, but no evidences were found. Due to the good validation results for this analyte, precision and accuracy in the required ranges, *R*^2^ > 0.99, selectivity and specificity given, we did not cancel it from the compound list in the routine analysis but referred to the sand and blank samples in every batch of routine analysis. Thus, concentrations of metribuzin DADK in real samples were only reported when higher than sand and RFNo (blank) samples.

In summary, QA/QC for both, SR and RFNo were all within the SANTE guidelines (SANTE 2020, 2021). The comparable figures of merits (i.e., absolute and relative recoveries, *LOQ*, spans of the 1^st^ to the 3^rd^ quantiles of ME for compounds with si and nsi IL-IS) obtained for RFN (i.e. the most common, though frequently pesticide contaminated, and agricultural soil type) and SR (a Cuban soil-type free of pesticides) lend credit to our decision to use the latter for method validation.

#### Routine analysis

Based on the validation results and quality control presented above, our method proofed fit-for-purpose for 38 analytes. These were monitored in agricultural soils for potato production from Mayabeque, Cuba. A total of 29 compounds were detected and quantified above the *LOQ* in 30 soil samples (Fig. 3, RFNc *n* = 26 and RFNo *n* = 4). Only four of these compounds presented recoveries < 60% or > 140% (Table S9), with good precision (*RSD*_*R*_ < 30%) at the lowest spiked level (10 ng/g). Their concentrations between *LOQ* and 10 ng/g were interpreted with care. However, all compound’s concentration values were kept and used (Fig. 3) as such for further data interpretation. Ninety percent of the soil samples had at least one CUP and/or TP residue in a range of 0.3–306 ng/g_dry weight (dw)_ (median = 10 ng/g_dw_). Pesticide residues most frequently detected (% in soil samples, min–max concentration, Fig. 3) were dicofol (73%, 0.8–35 ng/g_dw_), s-metolachlor (70%, 1.5–306 ng/g_dw_), dimethomorph (60%, 1.7–59 ng/g_dw_), and azoxystrobin (57%, 0.5–198 ng/g_dw_). Only three (fluopicolide, chlorothalonil, and dimethomorph) of the ten most frequently applied pesticides in potato in Cuba (mancozeb, chlorothalonil, potassium phosphite, propineb, dimethomorph, fluopicolide, propamocarb hydrochloride, valiphenalate, folpet, and glyphosate, (Barroso Planas 2014)) coincide with the investigated ai (marked with a “+” in Fig. 3). All soil samples of conventionally managed fields exhibited co-occurrence of three or more CUP and/or TP residues. In contrast, only two CUPs could be detected in one soil sample of the organically managed sites: chlorothalonil (3.9 ng/g_dw_) and azoxystrobin (8.1 ng/g_dw_). The residues detected and quantified in this study were magnitudes lower than reported by Tan et al. (2020) who quantified 41 pesticides in a tropical soil of China in a concentration range from not detected to 11.7 mg/kg, with a median of 0.20 mg/kg. The CUP concentrations in this study were also lower than the ones reported by Silva et al. (2019) in temperate soils from 11 countries of Europa (median = 0.15 mg/kg, maximum = 2.9 mg/kg). Additionally, the sum of co-occurring pesticides exceeded in 63% (*n* = 19) and 67% (*n* = 20) of soil samples the Czech limit (100 ng/g) (MoECR 1994) and the Netherlands limit (70 ng/g) (VROM 2006), respectively. Currently, soil guidance values are yet to be established in most of countries, including Cuba.

In summary, the here developed analytical method could be successfully applied to real soil samples to quantify CUPs and TPs from Cuban agroecosystems. This method will now be applied to a larger monitoring campaign where soils of agricultural fields with potato plants were sampled over 4 consecutive years. The results will be published in due course and time.

## Applicability of the method to other low-income countries

A QuEChERS extraction and GC-MS/MS based method was thoroughly validated for 31 CUPs and seven TPs in SR, a tropical soil from Cuba. Additionally, ME_ext/int_ were evaluated for five different Cuban soils. All figures of merit proved satisfying for all compounds. The method was successfully applied to 30 soil samples (all RFN) and demonstrated to be well suitable for monitoring pesticide residues in this matrix. We consider it fit to transfer to other laboratories, contributing to the establishment of soil monitoring programs in developing and tropical countries, in which studies are limited. Often, no MS is available in such labs but given the importance of internal standards, other, nsi, thermostable compounds with similar volatility as the analytes of interest could be used. Therefore, monitoring studies are crucial for future establishment of RGV in agricultural soils of tropical countries, including Cuba. Moreover, we present a solid research tool with less means than in high-income countries at comparably higher demanding environmental conditions, such as tropical humidity and temperature.

### Supplementary Information

Below is the link to the electronic supplementary material.

Supplementary file1 (PDF 1.74 MB)

## Data Availability

The datasets presented in this work are available from the corresponding author on reasonable request.

## References

[CR1] Acosta-Dacal A, Rial-Berriel C, Díaz-Díaz R, del Mar Bernal-Suárez M, Luzardo OP (2021). Optimization and validation of a QuEChERS-based method for the simultaneous environmental monitoring of 218 pesticide residues in clay loam soil. Sci Total Environ.

[CR2] Anastassiades M, Lehotay SJ, Štajnbaher D, Schenck FJ (2003). Fast and easy multiresidue method employing acetonitrile extraction/partitioning and “dispersive solid-phase extraction” for the determination of pesticide residues in produce. J AOAC Int.

[CR3] Anastassiades M, Maštovská K, Lehotay SJ (2003). Evaluation of analyte protectants to improve gas chromatographic analysis of pesticides. J Chromatogr A.

[CR4] Andrade JM, Estévez-Pérez MG (2014). Statistical comparison of the slopes of two regression lines: a tutorial. Anal Chim Acta.

[CR5] Asensio-Ramos M, Hernández-Borges J, Ravelo-Pérez L, Rodriguez-Delgado M (2010). Evaluation of a modified QuEChERS method for the extraction of pesticides from agricultural, ornamental and forestal soils. Anal Bioanal Chem.

[CR6] Barroso Planas K (2014) Tendencias en el uso de plaguicidas y agentes de control biológico en Solanum tuberosum L., en Cuba, Universidad Agraria de La Habana “Fructuoso Rodríguez Pérez” San José de las Lajas, p 48

[CR7] Bortolozo F, Aguiar TR, Hansel F, Rosa Filho E, Parron L, Froehner S (2016). Peatland as a natural sink for pesticides from no-till systems in subtropical climate. Agr Water Manag.

[CR8] Carvalho FP (2017). Pesticides, environment, and food safety. Food Energy Secur.

[CR9] de Souza RM, Seibert D, Quesada HB, de Jesus Bassetti F, Fagundes-Klen MR, Bergamasco R (2020). Occurrence, impacts and general aspects of pesticides in surface water: a review. Process Saf Environ.

[CR10] Dierksmeier G (1996). Pesticide contamination in the Cuban agricultural environment. Trend Anal Chem.

[CR11] EC (2010) SANCO/825/00 Rev. 8.1. Guidance document on pesticide residue analytical methods. 2010. European Commission. Directorate General Health and Consumer Protection. pp 1–28. Publishing European Commission. https://www.biotecnologiebt.it/download/SANCO_825_00_rev8_1_2010.pdf. Accessed 11 Apr 2023

[CR12] EC (2019) Commission implementing regulation (EU) 2019/533 concerning a coordinated multiannual control programme of the union for 2020, 2021 and 2022 to ensure compliance with maximum residue levels of pesticides and to assess the consumer exposure to pesticide residues in and on food of plant and animal origin. Off J Eur Union L88/28. Publishing Official Journal of the European Union. https://eur-lex.europa.eu/legal-content/EN/TXT/PDF/?uri=CELEX:32019R0533&from=DA. Accessed 18 July 2023

[CR13] FAO (2022) Pesticides use, pesticides trade and pesticides indicators - global, regional and country trends, 1990-2020. FAOSTAT Rome. Anal Briefs 46. Publishing Food and Agricultural Organization of United State. https://www.fao.org/3/cc0918en/cc0918en.pdf. Accessed 25 July 2023

[CR14] Febles-González J, Vega M, Sobrinho NA (2013) La degradación de los suelos Ferralíticos Rojos en el occidente de Cuba, I Seminario Internacional de Manejo Sostenible de suelos agrarios y de recursos naturales, Guaranda, pp 6–7. Publishing University of Almeria. https://www.researchgate.net/publication/327163998_LA_DEGRADACION_DE_LOS_SUELOS_FERRALITICOS_ROJOS_EN_EL_OCCIDENTE_DE_CUBA#fullTextFileContent. Accessed 20 Apr 2023

[CR15] García-Hernández J, Leyva-Morales JB, Bastidas-Bastidas PdJ, Leyva-García GN, Valdez-Torres JB, Aguilar-Zarate G, Betancourt-Lozano M (2021). A comparison of pesticide residues in soils from two highly technified agricultural valleys in northwestern Mexico. J Environ Sci Health, Part B.

[CR16] Geissen V, Silva V, Lwanga EH, Beriot N, Oostindie K, Bin Z, Pyne E, Busink S, Zomer P, Mol H (2021). Cocktails of pesticide residues in conventional and organic farming systems in Europe-Legacy of the past and turning point for the future. Environ Po.

[CR17] Guarda PM, Gualberto LdS, Mendes DB, Guarda EA, da Silva JEC (2020). Analysis of triazines, triazoles, and benzimidazoles used as pesticides in different environmental compartments of the Formoso River and their influence on biodiversity in Tocantins. J Environ Sci Health, Part B.

[CR18] Gubler A, Wächter D, Blum F, Bucheli TD (2015). Remarkably constant PAH concentrations in Swiss soils over the last 30 years. Environ Sci Process Impacts.

[CR19] Hartmann N, Erbs M, Wettstein FE, Schwarzenbach RP, Bucheli TD (2007). Quantification of estrogenic mycotoxins at the ng/L level in aqueous environmental samples using deuterated internal standards. J Chromatogr A.

[CR20] Homazava N, Gachet Aquillon C, Vermeirssen E, Werner I (2014). Simultaneous multi-residue pesticide analysis in soil samples with ultra-high-performance liquid chromatography–tandem mass spectrometry using QuEChERS and pressurised liquid extraction methods. Int J Environ Anal Chem.

[CR21] Horwitz W (1982). Evaluation of analytical methods used for regulation of foods and drugs. Anal Chem.

[CR22] Hvězdová M, Kosubová P, Košíková M, Scherr KE, Šimek Z, Brodský L, Šudoma M, Škulcová L, Sáňka M, Svobodová M, Krkošková L, Vašíčková J, Neuwirthová N, Bielská L, Hofman J (2018). Currently and recently used pesticides in Central European arable soils. Sci Total Environ.

[CR23] ISO (2013) Soil quality — determination of polychlorinated biphenyls (PCB) by gas chromatography with mass selective detection (GC-MS) and gas chromatography with electroncapture detection (GC-ECD), ISO, pp 2. Publishing International Standard Organization (ISO). https://www.iso.org/obp/ui/en/#iso:std:iso:13876:ed-1:v1:en. Accessed 14 June 2023

[CR24] Jennings AA, Li Z (2014). Scope of the worldwide effort to regulate pesticide contamination in surface soils. J Environ Manage.

[CR25] Kalyabina VP, Esimbekova EN, Kopylova KV, Kratasyuk VA (2021). Pesticides: formulants, distribution pathways and effects on human health–a review. Toxicol Rep.

[CR26] Kumari B, Madan V, Kathpal T (2008). Status of insecticide contamination of soil and water in Haryana, India. Environ Monit Assess.

[CR27] Lewis KA, Tzilivakis J, Warner DJ, Green A (2016). An international database for pesticide risk assessments and management. Hum Ecol Risk Assess: An Int J.

[CR28] Li Z (2022). New implication of pesticide regulatory management in soils: average vs ceiling legal limits. Sci Total Environ.

[CR29] Łozowicka B, Rutkowska E, Jankowska M (2017). Influence of QuEChERS modifications on recovery and matrix effect during the multi-residue pesticide analysis in soil by GC/MS/MS and GC/ECD/NPD. Environ Sci Pollut Res.

[CR30] Mahdavi V, Heris M-ES, Dastranj M, Farimani MM, Eslami Z, Aboul-Enein HY (2021). Assessment of pesticide residues in soils using a QuEChERS extraction procedure and LC-MS/MS. W AirSoil Pollut.

[CR31] Md Meftaul I, Venkateswarlu K, Dharmarajan R, Annamalai P, Megharaj M (2020). Pesticides in the urban environment: a potential threat that knocks at the door. Sci Total Environ.

[CR32] MINAG (2008) Lista Oficial de Plaguicidas Autorizados. Registro Central de Plaguicidas. República de Cuba. Publishing Registro Central de Plaguicidas. https://www.yumpu.com/es/document/read/47662266/lista-oficial-de-plaguicidas-autorizados. Accessed 16 May 2023

[CR33] MINAG (2016) Lista Oficial de Plaguicidas Autorizados. Registro Central de Plaguicidas. República de Cuba

[CR34] MINAG (2022) Lista Oficial de Plaguicidas Autorizados. Registro Central de Plaguicidas. República de Cuba. Publishing Registro Central de Plaguicidas. https://www.minag.gob.cu/wp-content/uploads/2023/06/Lista-Oficial-de-Plaguicidas.pdf. Accessed 14 Nov 2023

[CR35] MoECR (1994) Decree 13/1994. Specifying the Details of the Agricultural Soil Protection. http://www.mzp.cz/www/platnalegislativa.nsf. Accessed 20 Sept 2023

[CR36] Murugan A, Swarnam T, Gnanasambandan S (2013). Status and effect of pesticide residues in soils under different land uses of Andaman Islands, India. Environ Monit Assess.

[CR37] Padilla-Sánchez JA, Romero-González R, Plaza-Bolaños P, Garrido Frenich A, Martinez Vidal JL (2015). Residues and organic contaminants in agricultural soils in intensive agricultural areas of Spain: a three years survey. CLEAN–Soil. Air, W.

[CR38] Pérez-Consuegra N, Montano-Pérez M (2021) Los Plaguicidas Altamente Peligrosos en Cuba, Editora Agroecológica 56. Publishing IPEN/ACTAF/RAPAL. https://ipen.org/sites/default/files/documents/hhp_hhp_cuba_26_abril_2021_spanish_final_version.pdf. Accessed 6 Sept 2023

[CR39] PPDB (2023) Pesticide properties database, agriculture & environment research unit (AERU), University of Hertfordshire, UK, https://sitem.herts.ac.uk/aeru/ppdb/en/atoz.htm. Accessed 22 Mar 2023

[CR40] Primost JE, Marino DJ, Aparicio VC, Costa JL, Carriquiriborde P (2017). Glyphosate and AMPA, “pseudo-persistent” pollutants under real-world agricultural management practices in the Mesopotamic Pampas agroecosystem, Argentina. Environ Pollut.

[CR41] Riedo J, Wettstein FE, Rösch A, Herzog C, Banerjee S, Büchi L, Charles R, Wächter D, Martin-Laurent F, Bucheli TD (2021). Widespread occurrence of pesticides in organically managed agricultural soils—the ghost of a conventional agricultural past?. Environ Sci Technol.

[CR42] Riedo J, Wächter D, Gubler A, Wettstein FE, Meuli RG, Bucheli TD (2023). Pesticide residues in agricultural soils in light of their on-farm application history. Environ Pollut.

[CR43] Rösch A, Wettstein FE, Wächter D, Reininger V, Meuli RG, Bucheli TD (2023). A multi-residue method for trace analysis of pesticides in soils with special emphasis on rigorous quality control. Anal Bioanal Chem.

[CR44] Sabzevari S, Hofman J (2022). A worldwide review of currently used pesticides' monitoring in agricultural soils. Sci Total Environ.

[CR45] SANTE (2020) SANTE 12830/2020-guidance document on pesticide analytical methods for risk assessment and post-approval control and monitoring purposes. Supersedes Guidance Documents SANCO/3029/99 and SANCO/825/00. https://food.ec.europa.eu/document/download/022e0538-7fcd-4fa3-8b99-f3ba2afc4c3d_en?filename=pesticides_ppp_app-proc_guide_res_mrl-guidelines-2020-12830.pdf. Accessed 12 July 2023

[CR46] SANTE (2021) SANTE 11312/2021- Analytical quality control and method validation procedures for pesticide residues analysis in food and feed. Supersedes Document No. SANTE/2019/12682. https://www.accredia.it/app/uploads/2021/02/SANTE_11312_2021.pdf. Accessed 12 July 2023

[CR47] Seneff S (2021) Toxic legacy, how the weedkiller glyphosate is destroying our health and the environment, Vermont, USA

[CR48] Sidhu GK, Singh S, Kumar V, Dhanjal DS, Datta S, Singh J (2019). Toxicity, monitoring and biodegradation of organophosphate pesticides: a review. Crit Rev Env Sci Tec.

[CR49] Silva V, Mol HG, Zomer P, Tienstra M, Ritsema CJ, Geissen V (2019). Pesticide residues in European agricultural soils–a hidden reality unfolded. Sci Total Environ.

[CR50] Słowik-Borowiec M, Szpyrka E, Książek-Trela P, Podbielska M (2022). Simultaneous determination of multi-class pesticide residues and PAHs in plant material and soil samples using the optimized QuEChERS method and tandem mass Spectrometry Analysis. Molecules.

[CR51] Syafrudin M, Kristanti RA, Yuniarto A, Hadibarata T, Rhee J, Al-Onazi WA, Algarni TS, Almarri AH, Al-Mohaimeed AM (2021). Pesticides in drinking water—a review. Int J Env Res Pub He.

[CR52] Tan H, Li Q, Zhang H, Wu C, Zhao S, Deng X, Li Y (2020). Pesticide residues in agricultural topsoil from the Hainan tropical riverside basin: determination, distribution, and relationships with planting patterns and surface water. Sci Total Environ.

[CR53] Tudi M, Daniel Ruan H, Wang L, Lyu J, Sadler R, Connell D, Chu C, Phung DT (2021). Agriculture development, pesticide application and its impact on the environment. Int J Env Res Pub He.

[CR54] Valverde MG, Bueno MM, Gómez-Ramos M, Aguilera A, García MG, Fernández-Alba A (2021). Determination study of contaminants of emerging concern at trace levels in agricultural soil. A pilot study. Sci Total Environ.

[CR55] Velasco A, Rodríguez J, Castillo R, Ortíz I (2012). Residues of organochlorine and organophosphorus pesticides in sugarcane crop soils and river water. J Environ Sci Heal B.

[CR56] VROM (2006). Soil quality regulation.

[CR57] Vu-Duc N, Minh-Le T, Nguyen-Thi X, Vu CT, Bui V-H, Vu-Thi HA, Chu DB (2023) Analysis of organochlorine pesticides in contaminated soil by GC-MS/MS using accelerated solvent extraction as a green sample preparation. Int J Environ Anal Chem 1-12. 10.1080/03067319.2023.2264191

[CR58] Wang S, Hou Z, Liang S, Lu Z (2020). Residue behavior and risk assessment of rimsulfuron and quizalofop-p-ethyl in potato under field conditions. B Environ Contam Tox.

[CR59] WRB IWG (2015) World reference base for soil resources 2014, update 2015 International soil classification system for naming soils and creating legends for soil maps. Food and Agriculture Organization of the United Nations, Rome. World Soil Resources Reports No. 106. Publishing FAO. https://www.fao.org/3/i3794en/I3794en.pdf. Accessed 18 July 2023

[CR60] Zikankuba VL, Mwanyika G, Ntwenya JE, James A (2019). Pesticide regulations and their malpractice implications on food and environment safety. Cog Food Agric.

