# Supplementary material for: Validation of a modified QuEChERS method for the quantification of residues of currently used pesticides in Cuban agricultural soils, using gas chromatography tandem mass spectrometry
Source: Environ Sci Pollut Res Int. 2024 Apr 30;31(23):33623–37. doi: 10.1007/s11356-024-33237-6 (PMC11136849; doi:10.1007/s11356-024-33237-6)
Supplement: Supplementary file 1 — Supplementary file1 (PDF 1.74 MB) [file 11356_2024_33237_MOESM1_ESM.pdf]

Validation of a modified QuEChERS method for the quantification of residues of currently used pesticides in Cuban agricultural soils, using gas chromatography tandem mass spectrometry: Supporting Information

Brizeidi Peña[1], Dayana Sosa[1,2*], Isabel Hilber[2], Arturo Escobar[1], Thomas Daniel Bucheli[2]

Affiliation:

[1] Analytical Unit of Residues and Contaminants, National Center for Animal and Plant Health (CENSA), San José de las Lajas, P.O. Box 10, 32700 Mayabeque, Cuba

[2] Agroscope Environmental Analytics, Reckenholzstrasse 191, 8046 Zurich, Switzerland

***Corresponding author:**

Dayana Sosa, email: dayanasosap@gmail.com
ORCID: Dayana Sosa, orcid.org/0000-0002-8867-0492

Content

1 Pesticides, transformation products and isotopically labelled internal standards (IL-IS) used in the study ... 3
2 Sampling and soil characteristics. ... 7
3 Instrument conditions ... 9
3.1 Optimization of chromatographic and mass conditions ... 10
3.2 GC-MS/MS instrument conditions ... 11
3.3 Specificity and selectivity in SR ... 15
4 Method validation ... 22
4.1 Slope comparison of matrix matched calibrations with the internal standard method of the soils 22
4.2 Matrix effect in different soils ... 23
4.3 Figures of merit: Absolute recovery ... 33
5 Application of the method to real samples ... 34
5.1 Quality control and quality assurance (QA/QC) ... 34
6 Supplementary References ... 38

1 Pesticides, transformation products and isotopically labelled internal standards (IL-IS) used in the study

Table S1 Analytes included in the analytical method, numbered according to their retention time (Table S3), with CAS-Number, provider and purity as declared by the provider.

No.	Analytes	CAS-Nr.[a]	Provider	Purity (%)
**1**	metribuzin desamino diketo (DADK)	52236-30-3	LGC|Dr. Ehrenstorfer, Augsburg, Germany	99.00
**2**	atrazine desethyl	6190-65-4	LGC|Dr. Ehrenstorfer, Augsburg, Germany	99.32
**3**	2,6-dichlorobenzamid	2008-58-4	LGC|Dr. Ehrenstorfer, Augsburg, Germany	99.43
**4**	atrazine	1912-24-9	LGC|Dr. Ehrenstorfer, Augsburg, Germany	99.08
**5**	clomazone	81777-89-1	LGC|Dr. Ehrenstorfer, Augsburg, Germany	99.69
**6**	chlorothalonil	1897-45-6	Sigma-Aldrich, Saint Louis, USA	99.7
**7**	metribuzin desamino (DA)	35045-02-4	LGC|Dr. Ehrenstorfer, Augsburg, Germany	99.83
**8**	pirimicarb	23103-98-2	LGC|Dr. Ehrenstorfer, Augsburg, Germany	99.57
**9**	metribuzin	21087-64-9	Sigma-Aldrich, Saint Louis, USA	≥99.0
**10**	ametryn	834-12-8	Sigma-Aldrich, Saint Louis, USA	98.6
**11**	metalaxyl	57837-19-1	LGC|Dr. Ehrenstorfer, Augsburg, Germany	99.34
**12**	prosulfocarb	52888-80-9	LGC|Dr. Ehrenstorfer, Augsburg, Germany	99.08
**13**	s-metolachlor	87392-12-9	LGC|Dr. Ehrenstorfer, Augsburg, Germany	98.27
**14**	dicofol	115-32-2	Sigma-Aldrich, Saint Louis, USA	99.5
**15**	trifloxystrobin CGA	252913-85-2	Sigma-Aldrich, Saint Louis, USA	99.7
**16**	triadimenol	55219-65-3	Sigma-Aldrich, Saint Louis, USA	98.4
**17**	α-endosulfane	115-29-7	Sigma-Aldrich, Saint Louis, USA	98.35
**18**	oxyfluorfen	42874-03-3	Sigma-Aldrich, Saint Louis, USA	99.7
**19**	fluazifop-p-butyl	69806-50-4	LGC|Dr. Ehrenstorfer, Augsburg, Germany	97.04
**20**	cyproconazole	94361-06-5	LGC|Dr. Ehrenstorfer, Augsburg, Germany	98.54
**21**	β-endosulfane	115-29-7	Sigma-Aldrich, Saint Louis, USA	98.35
**22**	carfentrazone-ethyl	128639-02-1	LGC|Dr. Ehrenstorfer, Augsburg, Germany	93.11
**23**	trifloxystrobin	141517-21-7	LGC|Dr. Ehrenstorfer, Augsburg, Germany	99.3
**24**	benalaxyl	71626-11-4	Sigma-Aldrich, Saint Louis, USA	100
**25**	fluopicolide	239110-15-7	LGC|Dr. Ehrenstorfer, Augsburg, Germany	99.0
**26**	endosulfane sulphate	1031-07-8	Sigma-Aldrich, Saint Louis, USA	99.7
**27**	tebuconazole	107534-96-3	LGC|Dr. Ehrenstorfer, Augsburg, Germany	98.6
**28**	epoxiconazole	135319-73-2	Sigma-Aldrich, Saint Louis, USA	98.0
**29**	bifenthrin	82657-04-3	Sigma-Aldrich, Saint Louis, USA	99.1
**30**	fenamidone	161326-34-7	Sigma-Aldrich, Saint Louis, USA	≥98.0
**31**	benthiavalicarb-isopropyl	177406-68-7	Sigma-Aldrich, Saint Louis, USA	≥98.0
**32**	fenamidone RPA 410193	332855-88-6	Sigma-Aldrich, Saint Louis, USA	98.9
**33**	pyraclostrobin	175013-18-0	Sigma-Aldrich, Saint Louis, USA	99.9
**34**	spirotetramat	203313-25-1	Sigma-Aldrich, Saint Louis, USA	≥98.0
**35**	boscalid	188425-85-6	LGC|Dr. Ehrenstorfer, Augsburg, Germany	99.02
**36**	deltamethrin	52918-63-5	Sigma-Aldrich, Saint Louis, USA	98.6

**37**	azoxystrobin	131860-33-8	LGC|Dr. Ehrenstorfer, Augsburg, Germany	98.72
**38**	dimethomorph	110488-70-5	LGC|Dr. Ehrenstorfer, Augsburg, Germany	98.59
**Group**	**IL-IS**			
**1**	atrazine desethyl-D7	1216649-31-8	CDN Isotopes Inc., Pointe-Claire, Canada	n.a.
**2**	2,6-dichlorobenzamid-D3	1219804-28-0	CDN Isotopes Inc., Pointe-Claire, Canada	n.a.
**3**	atrazine-D5	163165-75-1	CDN Isotopes Inc., Pointe-Claire, Canada	n.a.
**4**	n-methyl-metribuzin-D3	1794754-27-0	TRC, Toronto, Canada	n.a.
**5**	metribuzin-D3	n.a.	Sigma-Aldrich, Saint Louis, USA	≥98.0
**6**	metalaxyl-D6	1398112-32-7	CDN Isotopes Inc., Pointe-Claire, Canada	n.a.
**7**	s-metolachlor-D11	1632119-30-2	Sigma-Aldrich, Saint Louis, USA	≥97.0
**8**	trifloxystrobin-D6	n.a.	TRC, Toronto, Canada	n.a.
**9**	fluopicolide-D3	n.a.	HPC Standards GmbH, Cunnersdorf, Germany	n.a.
**10**	azoxystrobin-D4	1346606-39-0	TRC, Toronto, Canada	n.a.
	**Syringe standard**			
**-**	triphenyl phosphate (TPP)	115-86-6	Sigma-Aldrich, Saint Louis, USA	≥99.0

[a] n.a. = not available

Table S2 Properties of analyzed compounds including pesticide type, chemical class and formula, molecular weight, and logarithmized octanol-water partition coefficient (logK_{OW}). Compounds are listed according to their retention time (Table S3) and empty cells indicate information not available in the Pesticide Properties Database (PPDB) (PPDB 2023).

No.	Analytes	Type[c]	Chemical class and TP of No. x parent	Chemical formula	Molecular weight [amu]	Log K_{OW}
**1**	metribuzin DADK	TP	of no. 9	$C_7H_{11}N_3O_2$	169.2	1.49
**2**	atrazine desethyl	TP	of no. 4	$C_6H_{10}ClN_5$	187.6	1.51
**3**	2,6-dichlorobenz-amid	TP	of no. 25	$C_7H_5Cl_2NO$	190.3	0.38
**4**	atrazine	H	triazine	$C_8H1_4ClN_5$	215.6	2.70
**5**	clomazone	H	oxazole	$C_{12}H_{14}ClNO_2$	239.7	2.58
**6**	chlorothalonil	F	chloronitrile	$C_8Cl_4N_2$	265.9	2.94
**7**	metribuzin DA	TP	of no. 9	$C_8H_{13}N_3OS$	199.0	
**8**	pirimicarb	I/A	carbamate	$C_{11}H_{18}N_4O_2$	238.4	1.70
**9**	metribuzin	H	triazinona	$C_8H_{14}N_4OS$	214.3	1.75
**10**	ametryn	H	triazine	$C_9H_{17}N_5S$	227.1	2.63
**11**	metalaxyl	F	anilide/acrylamino acid	$C_{15}H_{21}NO_4$	279.3	1.75
**12**	prosulfocarb[a]	H	thiocarbamate	$C_{14}H_{21}NOS$	251.4	4.48
**13**	s-metolachlor	H	chloroacetamide	$C_{15}H_{22}ClNO_2$	283.8	3.05
**14**	dicofol	I/A	organochloride	$C_{14}H_9Cl_5O$	370.5	4.30
**15**	trifloxystrobin CGA	TP	of no. 23	$C_{19}H_{17}F_3N_2O_4$	394.0	
**16**	triadimenol	F	triazole	$C_{14}H_{18}ClN_3O_2$	295.8	3.18
**17**	α-endosulfane[b]	I/A	organochloride	$C_9H_6CL_6O_3S$	406.9	4.74
**18**	oxyfluorfen	H	nitrophenol ether	$C_{15}H_{11}ClF_3NO_4$	361.7	4.86
**19**	fluazifop-p-butyl	H	aryloxyphenoxypropionate	$C_{19}H_{20}F_3NO_4$	383.4	4.50
**20**	cyproconazole	F	triazole	$C_{15}H_{18}ClN_3O$	291.8	3.09
**21**	β-endosulfane [b]	I/A	organochloride	$C_9H_6Cl_6O_3S$	406.9	3.83
**22**	carfentrazone-ethyl [a]	H	triazolone	$C_{13}H_{14}Cl_2F_3N_3O_3$	412.2	3.70
**23**	trifloxystrobin	F	strobilurin	$C_{20}H_{19}F_3N_2O_4$	408.4	4.50
**24**	benalaxyl	F	fenilamine	$C_{20}H_{23}NO_3$	325.4	3.54
**25**	fluopicolide	F	benzamide	$C_{14}H_8Cl_3F_3N_2O$	383.6	2.90
**26**	endosulfane sulphate	TP	of no. 17 and 21	$C_9H_6Cl_6O_4S$	422.9	3.66
**27**	tebuconazole	F	triazole	$C_{16}H_{22}ClN_3O$	307.8	3.70
**28**	epoxiconazole	F	triazole	$C_{17}H_{13}ClFN_3O$	329.8	3.30
**29**	bifenthrin	I/A	pyrethroid	$C_{23}H_{22}ClF_3O_2$	422.9	6.60
**30**	fenamidone	F	imidazole	$C_{17}H_{17}N_3OS$	311.4	2.80
**31**	benthiavalicarb-isopropyl	F	carbamate	$C_{18}H_{24}FN_3O_3S$	381.5	2.56
**32**	fenamidone RPA	TP	of no. 30	$C_{16}H_{15}N_3O_2$	281.3	
**33**	pyraclostrobin	F	strobilurin	$C_{19}H_{18}ClN_3O_4$	387.8	3.99
**34**	spirotetramat	I/A	tetramic acid	$C_{21}H_{27}NO_5$	373.5	2.51

**35**	boscalid	F	carboxamide	$C_{18}H_{12}Cl_2N_2O$	343.2	2.96
**36**	deltamethrin	I/A	pyrethroide	$C_{22}H_{19}Br_2NO_3$	505.2	4.60
**37**	azoxystrobin	F	strobilurin	$C_{22}H_{17}N_3O_5$	403.4	2.50
**38**	dimethomorph	F	morpholine	$C_{21}H_{22}ClNO_4$	387.9	2.68

[a] not used or approved in Cuba according to the Official List of Pesticides of the Cuban Republic (2016) (MINAG 2008).

[b] Compounds banned in Cuba since 2013 (Pérez-Consuegra &Montano-Pérez 2021), but appear in the Official List of Pesticides of the Cuban Republic (MINAG 2008).

[c] Type of pesticide: I/A: insecticide/acaricide, F: fungicide, H: herbicide, TP: transformation product

2 Sampling and soil characteristics.

Table S3 Characteristics of soil sampling sites.

Soil sample number	Place	Management type	Soil type[a]	Use	Sampling date	Lat (o)	Lon (o)
**1[b]**	Jaruco	natural park	Skeletic regosol	forest	06/03/2014	23.04273	-82.06844
**2[b,c]**	San José de las Lajas	organic	Rhodic Ferralic Nitisol	potato	23/03/2020	23.011172	-82.1392
**3[b]**	Jaruco	conventional	Rhodic Ferralic Nitisol	agriculture	05/03/2014	23.04973	-81.93001
**4[b]**	San José de las Lajas	no management	Xanthic Ferralic Nitisol	forest	26/03/2014	22.93561	-82.08528
**5[b]**	Jaruco	conventional	Dystric Cambisol	livestodk	27/02/2014	23.06790	-28.05685
**6[c]**	Batabanó	conventional	Rhodic Ferralic Nitisol	potato	06/04/2021	22.773056	-82.319427
**7[c]**	Quivicán	conventional	Rhodic Ferralic Nitisol	potato	29/03/2021	22.87529	-82.39731
**8[c]**	Quivicán	conventional	Rhodic Ferralic Nitisol	potato	25/03/2021	22.85835	-82.39561
**9[c]**	Batabanó	conventional	Rhodic Ferralic Nitisol	potato	24/03/2021	22.77891	-82.25921
**10c**	San José de las Lajas	conventional	Rhodic Ferralic Nitisol	potato	25/03/2020	22.98551	-82.13493
**8[c]**	Quivicán	conventional	Rhodic Ferralic Nitisol	potato	24/03/2020	22.85835	-82.39561
**11[c]**	Batabanó	conventional	Rhodic Ferralic Nitisol	potato	10/03/2020	22.74835	-82.25058
**10[c]**	San José de las Lajas	conventional	Rhodic Ferralic Nitisol	potato	04/03/2020	22.98551	-82.13493
**12[c]**	Quivicán	conventional	Rhodic Ferralic Nitisol	potato	03/03/2020	22.76337	-82.3641
**7[c]**	Quivicán	conventional	Rhodic Ferralic Nitisol	potato	19/02/2020	22.87529	-82.39731
**2[c]**	San José de las Lajas	organic	Rhodic Ferralic Nitisol	potato	03/12/2019	23.011172	-82.1392
**13[c]**	Quivicán	conventional	Rhodic Ferralic Nitisol	potato	02/12/2019	22.81494	-82.456283
**12[c]**	Quivicán	conventional	Rhodic Ferralic Nitisol	potato	25/03/2019	22.76337	-82.3641
**7[c]**	Quivicán	conventional	Rhodic Ferralic Nitisol	potato	18/03/2019	22.87529	-82.39731
**13[c]**	Quivicán	conventional	Rhodic Ferralic Nitisol	potato	18/03/2019	22.81494	-82.456283
**15[c]**	Batabanó	conventional	Rhodic Ferralic Nitisol	potato	15/03/2019	22.793371	-82.26248
**16[c]**	Batabanó	conventional	Rhodic Ferralic Nitisol	potato	14/03/2019	22.75345	-82.27111
**2[c]**	San José de las Lajas	organic	Rhodic Ferralic Nitisol	potato	22/02/2019	23.011172	-82.1392
**17[c]**	Batabanó	conventional	Rhodic Ferralic Nitisol	potato	12/02/2019	22.72958	-82.256

**11**[c]	Batabanó	conventional	Rhodic Ferralic Nitisol	potato	07/02/2019	22.74835	-82.25058
**12**[c]	Quivicán	conventional	Rhodic Ferralic Nitisol	potato	01/02/2019	22.76337	-82.3641
**9**[c]	Batabanó	conventional	Rhodic Ferralic Nitisol	potato	08/01/2019	22.77891	-82.25921
**14**[c]	Quivicán	conventional	Rhodic Ferralic Nitisol	potato	04/01/2019	22.81494	-82.456283
**16**[c]	Batabanó	conventional	Rhodic Ferralic Nitisol	potato	14/12/2018	22.75345	-82.27111
**18**[c]	Quivicán	conventional	Rhodic Ferralic Nitisol	potato	05/12/2018	22.82806	-82.38035
**13**[c]	Quivicán	conventional	Rhodic Ferralic Nitisol	potato	30/11/2018	22.81494	-82.456283
**11**[c]	Batabanó	conventional	Rhodic Ferralic Nitisol	potato	22/11/2018	22.74835	-82.25058
**2**[c]	San José de las Lajas	organic	Rhodic Ferralic Nitisol	potato	15/11/2018	23.011172	-82.1392
**17**[c]	Batabanó	conventional	Rhodic Ferralic Nitisol	potato	09/11/2018	22.72958	-82.256

[a] Soil type terminology according to World Reference Base (WRB 2015), [b] Sites included in the ME study, [c] Sites included in the routine analysis

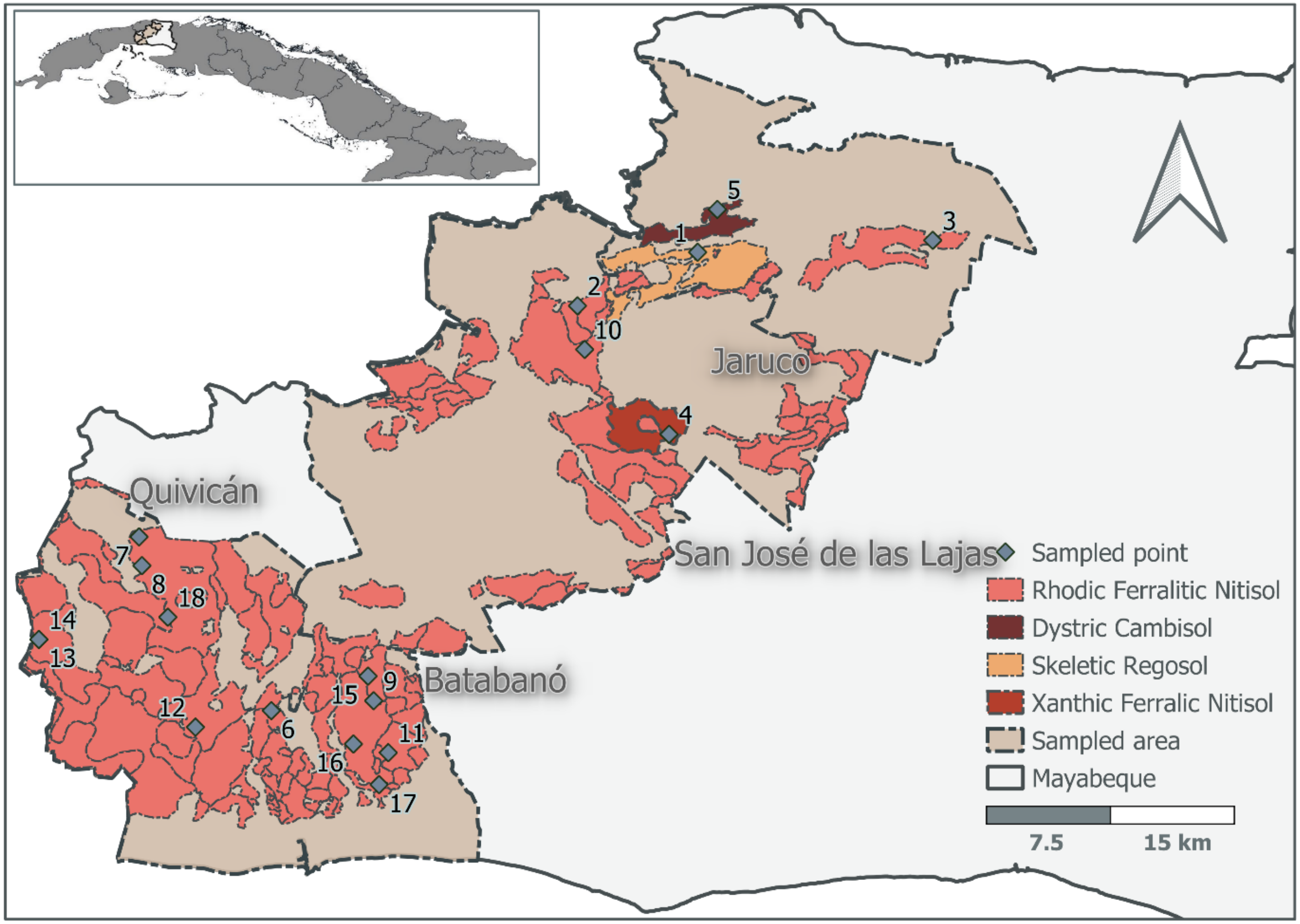

Fig S1 Soil was sampled in four different counties: Jaruco (JC), San José de las Lajas (SJ), Batabanó (BT) and Quivicán (QV) in the province of Mayabeque, Cuba. Individual soil sampling sites are marked with a grey diamond. Soils where the matrix effect was studied were from sites 1 to 5 and those used in the routine analysis were from sites 2, 6 to18.

3 Instrument conditions

3.1 Optimization of chromatographic and mass conditions

The injection volume was 0.5 µL due to the large vaporization volume of ACN and the little volume of the glass insert. To achieve the highest possible sensitivity, the injection mode was not split but splitless, which is the suggested injection mode by Shimadzu (manual for GCMS TQ series, Jul 2018, 225-38192) when concentrations are expected to be less than 10 ng. The TPP syringe standard was used not only to calculate absolute recovery (see chapter 2.7.2) but also for compensating possible variations of the small injection volume. Analytes were injected with protective agents such as sugars and acids (i.e., acetic acid and FA) (Anastassiades et al. 2003) to minimize peak distortion and compensate for matrix effects (ME). The use of ACN in combination with FA has previously been reported increasing sensitivity and improving peak shape in GC-MS/MS (Acosta-Dacal et al. 2021), and did here as well for most compounds.

Initially, pure compound solutions were injected and scanned in Q3 (full scan) to find the most abundant m/z parent ion. Three to five parent ions per compound were selected manually to run the product ion scans with different collision energies (CE) of up to 45 V in incremental steps of 3 V to find the optimal multiple reaction monitoring (MRM) transitions. Best or most abundant MRM were found with the MRM optimization tool of Shimadzu. Most compounds, thus native analytes, IL-IS and the syringe standard had several suitable MRM (Q1/Q3) transitions, the most sensitive of which we choose as quantifier. For twenty-eight compounds we could afford to monitor two qualifier ion transitions and in total 126 MRM transitions were used (Table S4). The optimal loop (in s) and processing times (in min) needed to be found. Shimadzu recommends 0.3 s and 0.3 min, respectively, but with that many events, MRM transitions of an individual analyte appeared up to four times in four different time windows. This redundancy unnecessarily decreased the dwell time. Hence, loop and processing time were put to the minimum defined by Shimadzu, which was 0.1 s and 0.1 min, and dwell time resulted in 2.5 to 48.5 ms with a median of 5.7 ms. Some peaks had a minimum number of points (9 – 10) due to the small dwell time, therefore a prolonged GC temperature program was tested according to Acosta-Dacal et al. (2021) from 21.3 to 37.4 min (80 °C with 1.5 min hold time, 20 °C/min to 190 °C and 5 °C/min to 230 °C, 25 °C/min to 290 °C, and hold for 20 min). Peaks revealed a better shape for some compounds but concomitantly, they also broadened (Fig. S2), which was why the short run was kept (for final retention times, see Table S4; please note when comparing short run retention times of Fig. S2 with Table S4 that these were subject to changes under routine operation). Interface and ion source were varied from 300 °C, each, to 250 °C and 200 °C, respectively, but peak heights of all compounds were lower with the lower temperatures except for the ones around 7 ± 0.5 min (Fig. S3).

3.2 GC-MS/MS instrument conditions

Table S4 Optimized GC-MS/MS conditions for all target analytes, isotopically labelled internal standards (IL-IS) and syringe standard. The column "IL-IS group" indicates the number of the corresponding IL-IS with which the compound was quantified. Empty cells in the last two columns mean that only one qualifier ion transition (and collision energy, CE) was used. The IL-IS group numbers are listed in Table S1.

No.	Compound Name	IL-IS group	Retention time (min)	Start Time (min)	End Time (min)	Event Time (sec)	Quantifier ion transition	CE	Qualifier ion 1 transition	CE	Qualifier ion 2 transition	CE
**1**	metribuzin DADK	4	6.701	6.66	7.10	0.100	154.0>83.0	12	154.0>111.1	6	111.0>83.1	6
**2**	atrazine desethyl	1	7.392	7.10	7.68	0.025	187.0>172.1	3	172.0>69.0	21	145.0>110.1	6
**3**	2,6-dichlorobenz-amid	2	7.465	7.10	7.68	0.025	189.0>173.0	9	173.0>144.9	15	173.0>109.0	27
**4**	Atrazine	1	7.795	7.68	8.13	0.027	215.0>58.0	9	215.0>173.0	6	200.0>104.0	18
**5**	Clomazone	1	7.857	7.68	8.13	0.027	204.0>107.1	21	125.0>89.1	15	125.0>99.1	18
**6**	Chlorothalonil	4	8.199	8.13	8.45	0.021	264.0>168.0	24	264.0>228.9	18	266.0>230.9	18
**7**	metribuzin DA	4	8.240	8.13	8.45	0.013	184.0>68.1	24	184.0>89.1	6		
**8**	Pirimicarb	5	8.398	8.13	8.45	0.013	166.0>71.2	21	166.0 >55.1	12		
**9**	Metribuzin	5	8.399	8.13	8.61	0.020	198.0>82.2	18	198.0>55.1	33	198.0>110.3	12
**10**	Ametryn	5	8.505	8.45	8.77	0.016	227.0>185.1	6	227.0>170.1	12	227.0>58.1	9
**11**	Metalaxyl	6	8.554	8.45	8.77	0.011	160.0>144.0	24	160.0>130.2	18		
**12**	Prosulfocarb	6	8.588	8.45	8.77	0.011	251.0>128.1	6	160.0>100.2	9		
**13**	s-metolachlor	7	8.840	8.77	9.09	0.029	162.0>117.1	30	162.0>91.1	27		
**14**	Dicofol	7	8.916	8.77	9.09	0.042	139.0>111.0	15	139.0>75.1	30	141>113	18
**15**	trifloxystrobin CGA	8	9.184	9.09	9.44	0.050	116.0>89.1	18	116.0>63.1	24		
**16**	Triadimenol	7	9.229	9.09	9.44	0.050	168.0>70.0	9	128.0>65.1	18		
**17**	α-endosulfane	7	9.525	9.44	9.98	0.026	205.0>170.1	6	239.0>143.0	39	207.0>172.2	3
**18**	Oxyfluorfen	7	9.623	9.44	9.98	0.026	252.0>195.0	30	252.0>146.2	30	252.0>170.0	24
**19**	fluazifop-p-butyl	7	9.727	9.44	9.98	0.016	282.0>91.2	18	282.0>238.1	18		
**20**	Cyproconazole	8	9.832	9.44	10.22	0.016	139.0>75.1	30	222.0>124.8	30		
**21**	β-endosulfane	7	9.977	9.44	10.22	0.016	160.0>125.0	18	207.0>171.9	9		
**22**	carfentrazone-ethyl	8	10.101	9.98	10.40	0.013	340.0>312.0	18	330.0>309.8	18	312.0>151.0	21
**23**	Trifloxystrobin	8	10.123	9.98	10.40	0.013	116.0>89.1	15	116.0>63.0	30	131.0>89.10	30

**24**	Benalaxyl	8	10.175	9.98	10.40	0.013	148.0>77.1	27	148.0>105.1	18	148.0>79.0	27
**25**	Fluopicolide	9	10.245	9.98	10.73	0.009	173.0>145.1	12	209.0>181.8	12		
**26**	endosulfane sulphate	9	10.298	10.22	10.73	0.012	272.0>236.6	21	274.0>238.9	24	272.0>140.6	36
**27**	Tebuconazole	9	10.359	10.22	10.73	0.012	250.0>125.0	12	125.0>89.1	18	125.0>98.9	18
**28**	Epoxiconazole	9	10.490	10.40	10.73	0.012	192.0>138.1	15	192.0>111.1	30	165.0>138.0	9
**29**	Bifenthrin	9	10.577	10.40	11.31	0.011	165.0>163.2	33	166.0>164.0	33		
**30**	Fenamidone	9	10.736	10.40	11.31	0.017	268.0>180.1	24	238.0>103.2	24	268.0>77.1	27
**31**	benthiavalicarb-isopropyl	9	10.921	10.73	11.31	0.027	116.0>98.0	6	180.0>127.1	18	116.0>55.0	15
**32**	fenamidone RPA	9	11.005	10.73	11.31	0.027	281.0>120.3	9	281.0>238.3	6	237.0>167.5	27
**33**	Pyraclostrobin	9	11.456	11.31	11.77	0.100	132.0>77.1	21	132.0>51.10	30		
**34**	Spirotetramat	9	11.890	11.77	12.18	0.100	286.0>216.1	21	214.0>171.1	9	300.0>268.3	6
**35**	Boscalid	10	12.247	11.77	13.01	0.100	342.0>140.1	21	342.0>112.1	33		
**36**	Deltamethrin	10	13.635	13.01	13.89	0.100	252.9>93.0	20	252.9>171.9	8		
**37**	Azoxystrobin	10	13.935	13.01	14.31	0.029	344.0>183.2	24	344.0>172.1	27		
**38**	Dimethomorph	10	14.025	13.89	14.31	0.028	301.0>165.1	18	165.0>136.9	12		
						**IL-IS**						
**1**	atrazine desethyl-D7	1	7.366	7.10	7.68	0.025	194.0>176.1	3	194.0>65.2	15	176.0>72.1	15
**2**	2,6-dichlorobenz-amid-D3	2	7.459	7.10	7.68	0.025	176.0>148.0	15	192.0>176.0	6	176.0>111.1	27
**3**	atrazine-D5	3	7.777	7.68	8.13	0.019	220.0>58.1	9	220.0>178.1	6		
**4**	*N*-methyl-metribuzin-D3	4	7.933	7.68	8.13	0.027	120.0>73.1	9	198.0>82.1	15	198.0>110.1	9
**5**	metribuzin-D3	5	8.383	8.13	8.61	0.020	201.0>82.0	15	201.0>55.1	24	201.0>110.0	6
**6**	metalaxyl-D6	6	8.532	8.45	8.77	0.016	138.0>120.1	12	212.0>152.0	6	212.0>168.1	6
**7**	s-metolachlor-D11	7	8.807	8.61	9.09	0.029	173.0>142.2	15	249.0>173.3	12		
**8**	trifloxystrobin-D6	8	10.111	9.98	10.4	0.009	137.0 >94.1	30	122.0>66.1	27		
**9**	fluopicolide-D3	9	10.239	9.98	10.4	0.013	178.0 >150.0	12	178.0 >113.0	18	178.0>111.0	21
**10**	azoxystrobin-D4	10	13.906	13.01	14.31	0.043	348.0 >333.1	18	348.0>156.1	36	348.0 >172.2	39
						**Syringe standard**						
**1**	Triphenyl phosphate (TPP)	11	10.374	10.22	73	0.008	215.0>168.1	15	169>115.1	30		

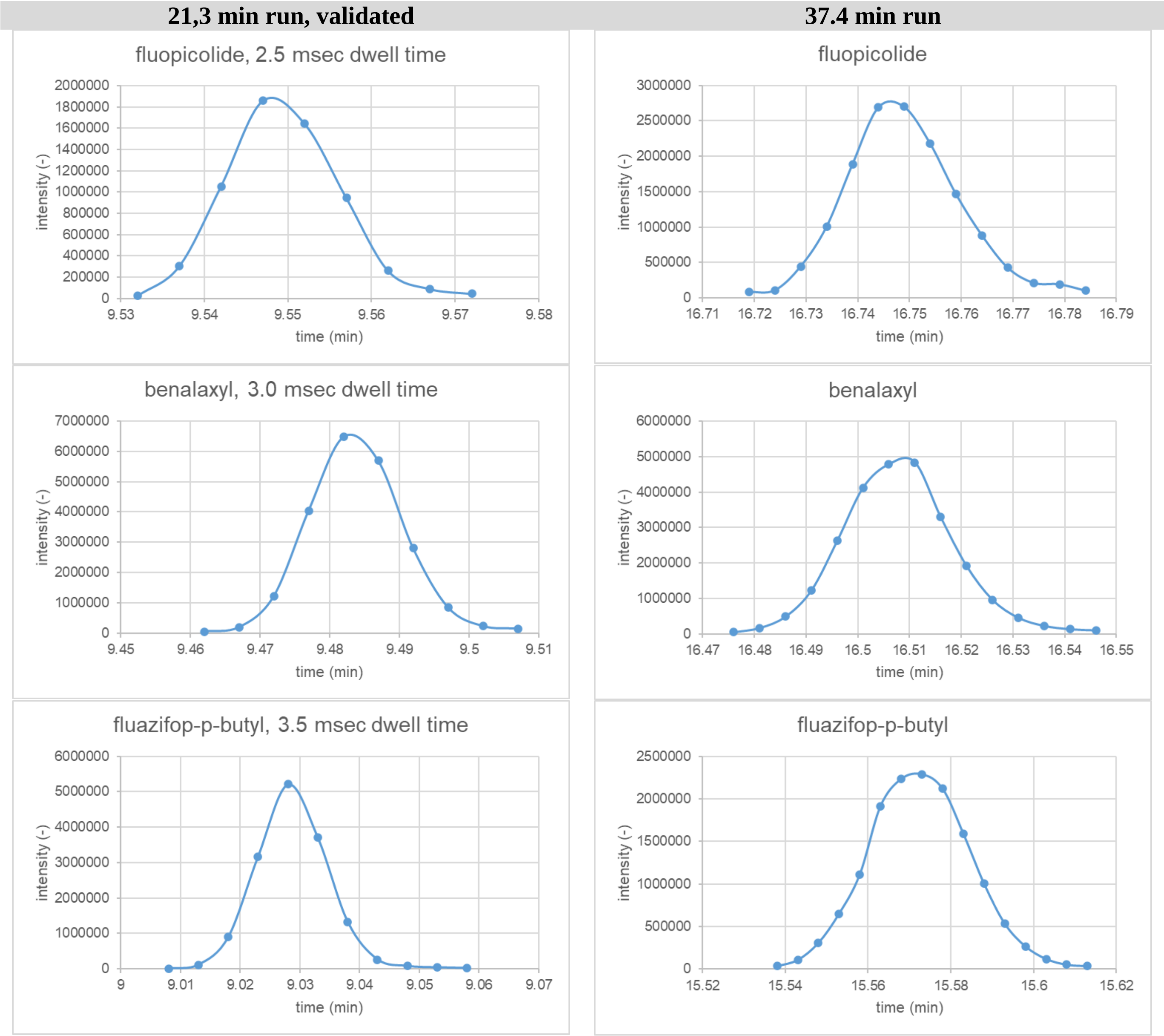

Fig S2 Chromatograms of 50 ng/mL concentrations of compounds with smallest dwell times in the short run (21.3 min, left column). The chromatograms of the long run (37.4 min) are depicted in the right column.

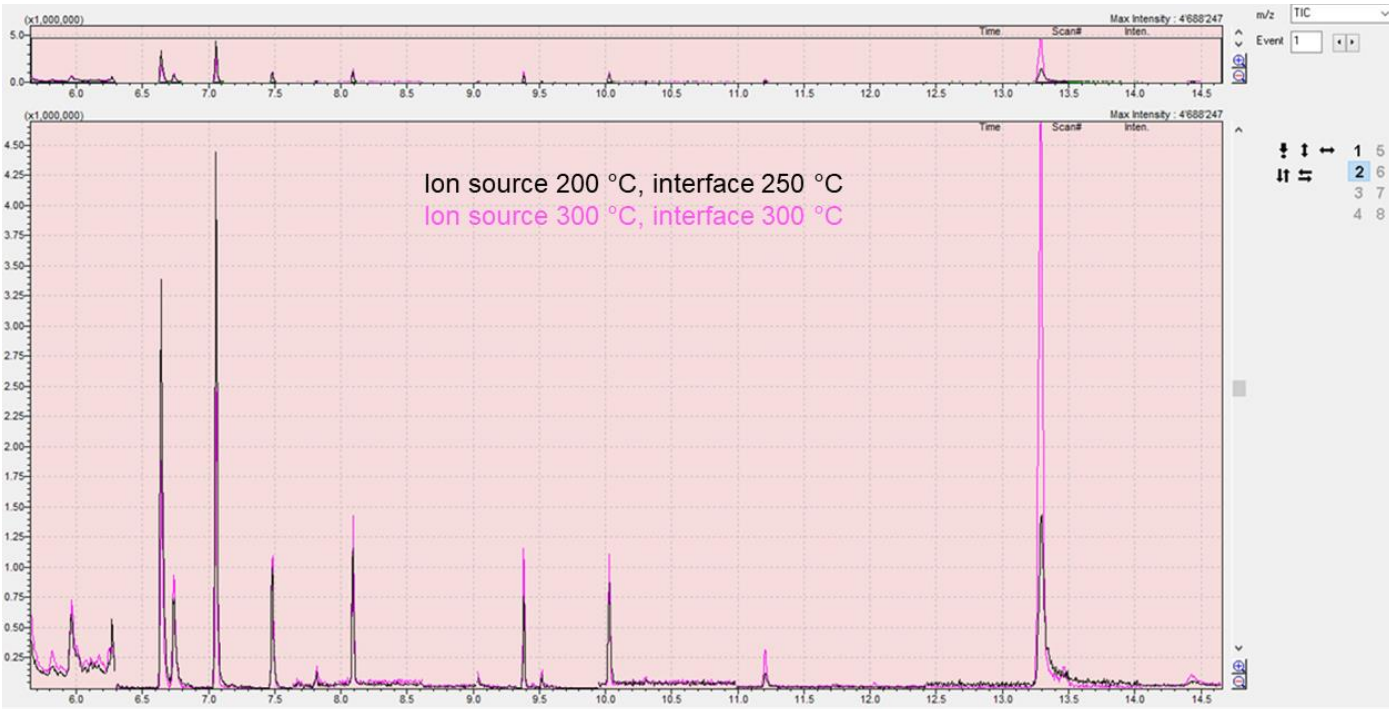

Fig S3 Chromatograms of 10 ng/mL standard mix with different interface and ion source temperatures.

3.3 Specificity and selectivity in SR

metribuzin DADK

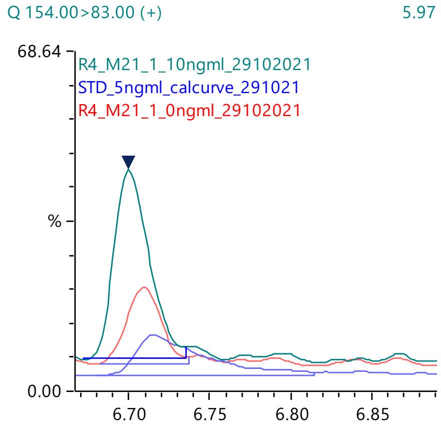

atrazine desethyl

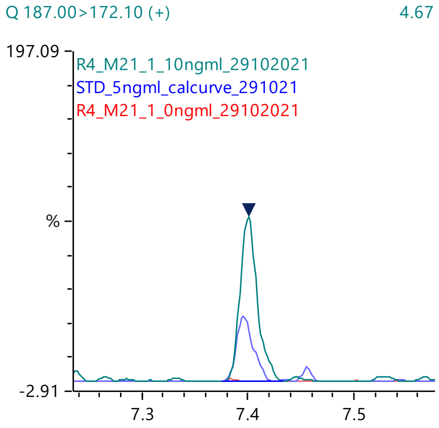

2.6-dichlorobenzamid

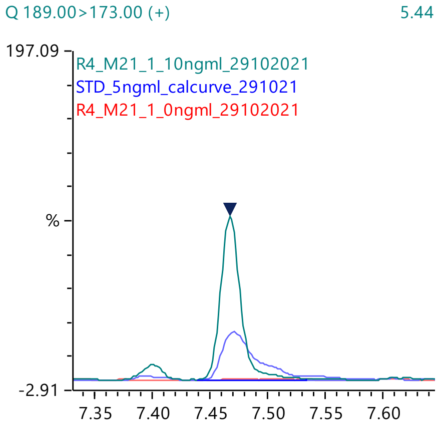

atrazine

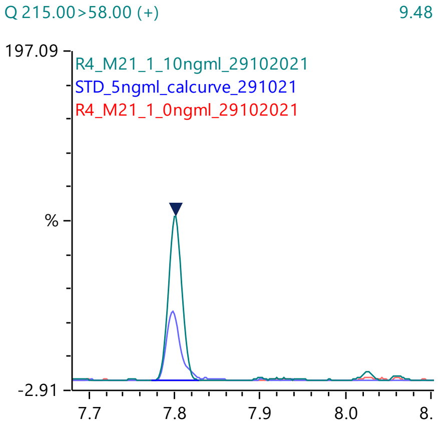

clomazone

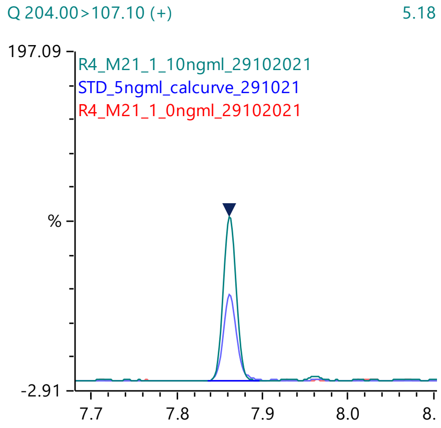

chlorothalonil

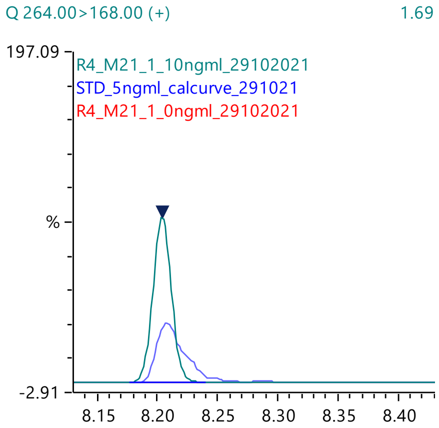

metribuzin DA

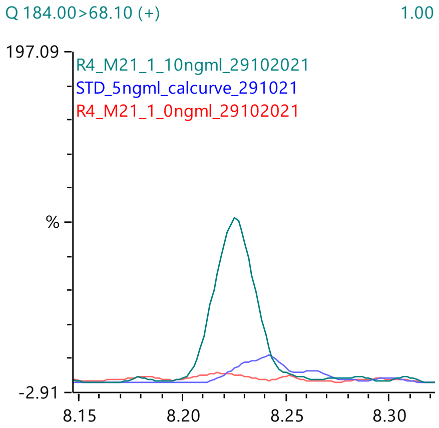

pirimicarb

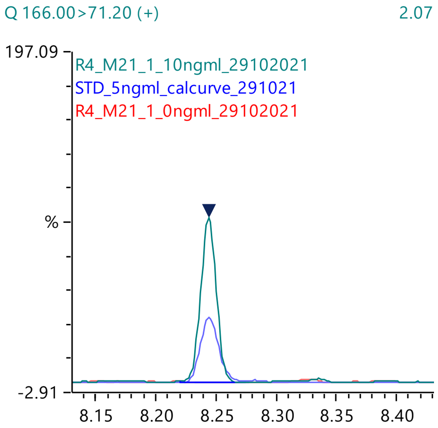

metribuzin

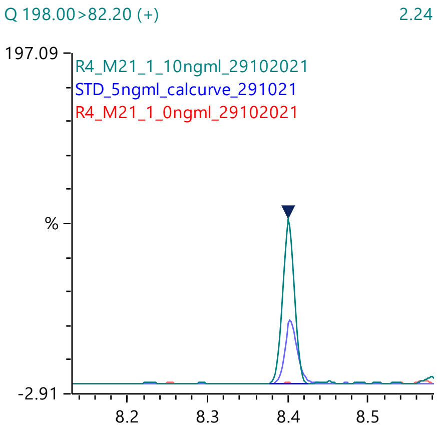

ametryn

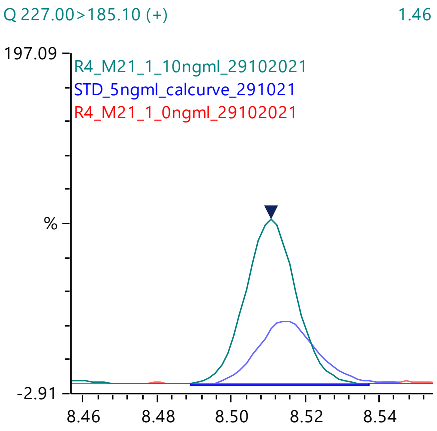

metalaxyl

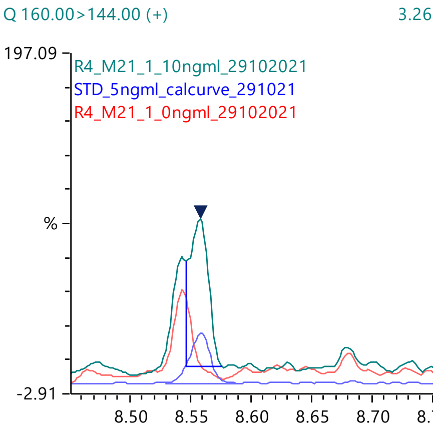

prosulfocarb

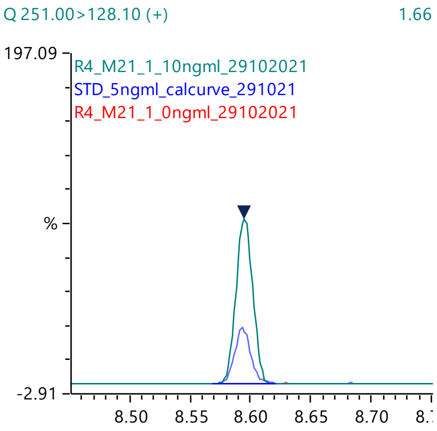

s-metolachlor

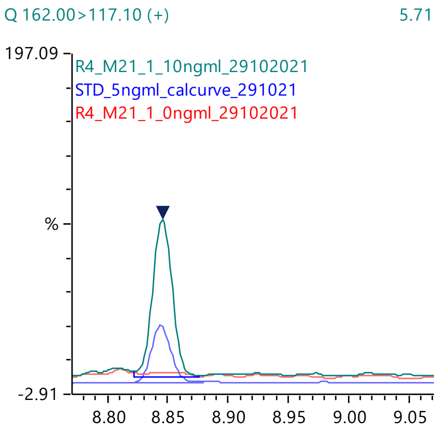

dicofol

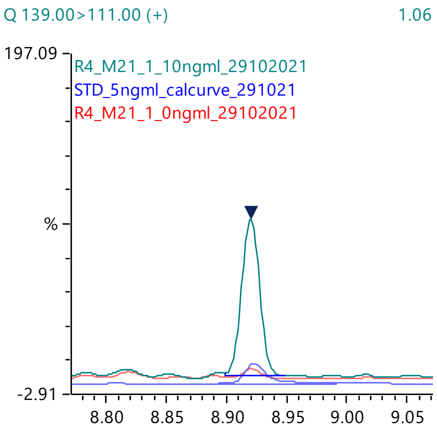

trifloxystrobin CGA

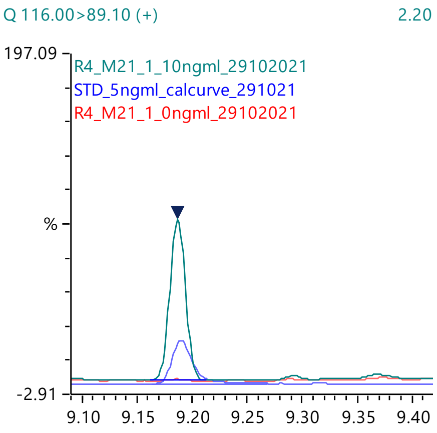

triadimenol

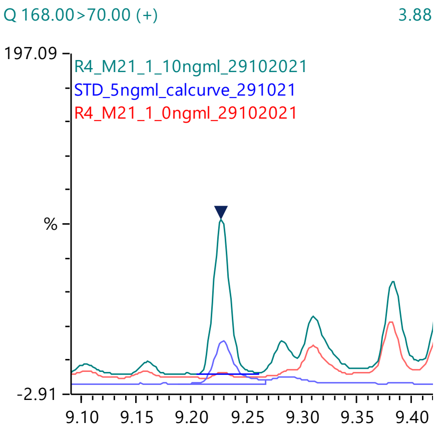

α-endosulfane

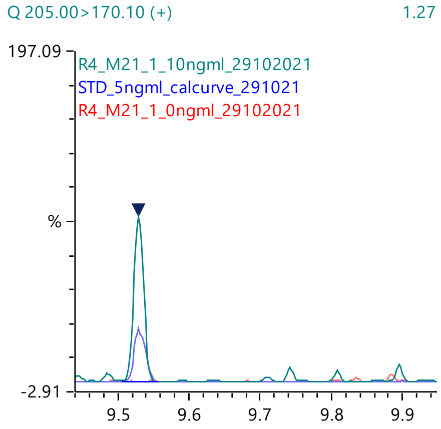

oxyfluorfen

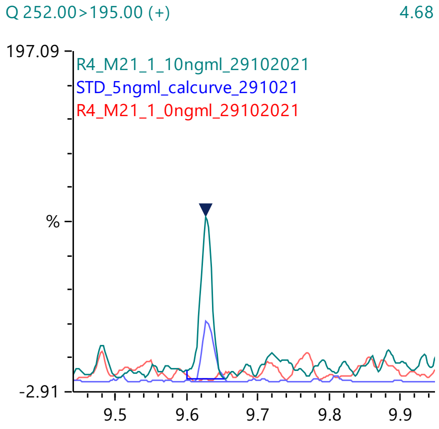

fluazifop-p-butyl

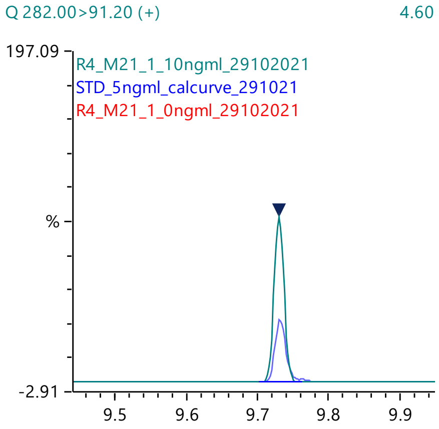

cyproconazole

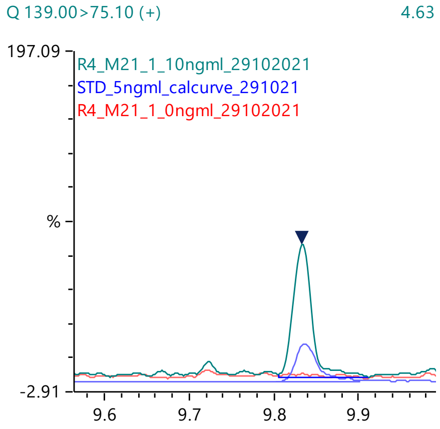

β-endosulfane

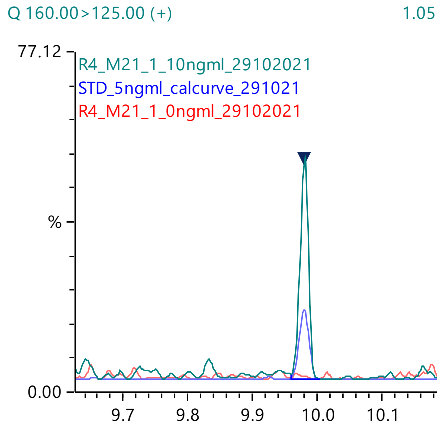

carfentrazone-ethyl

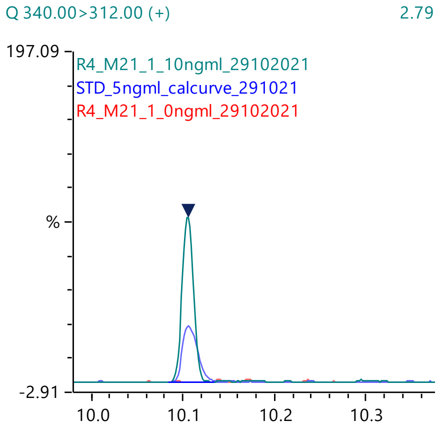

trifloxystrobin

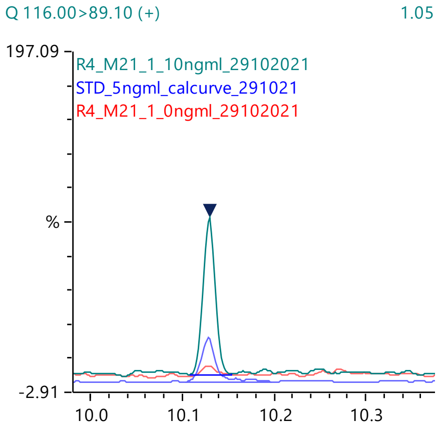

benalaxyl

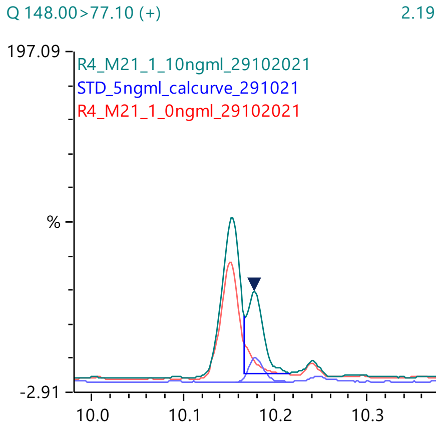

fluopicolide

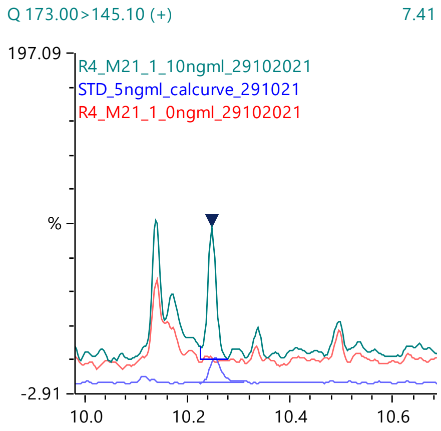

endosulfane sulphate

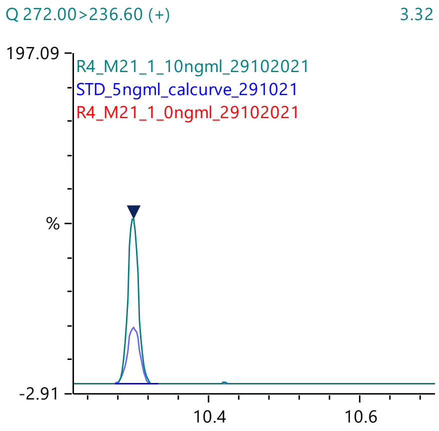

tebuconazole

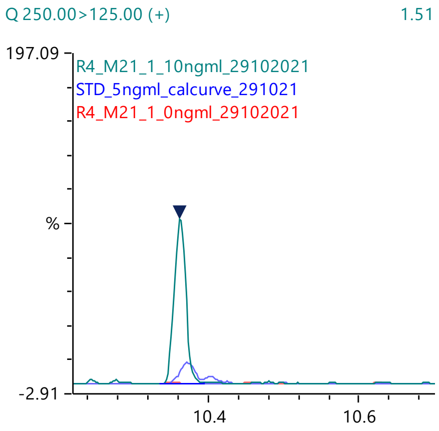

epoxiconazole

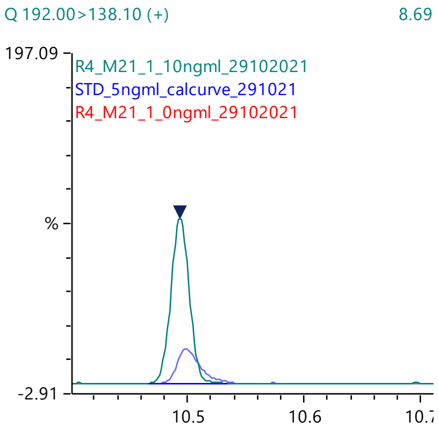

bifenthrin

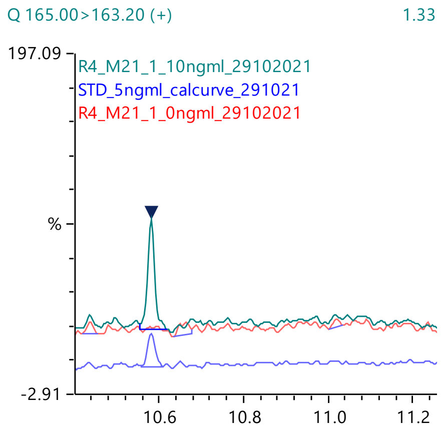

fenamidone

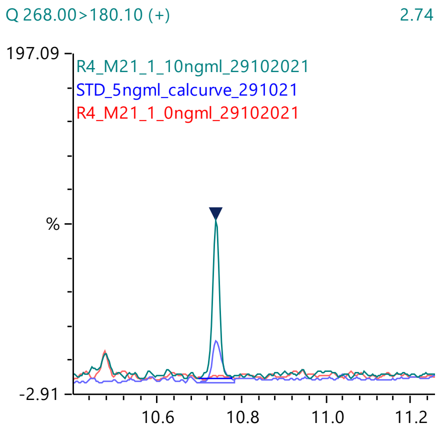

benthiavalicarb-isopropyl

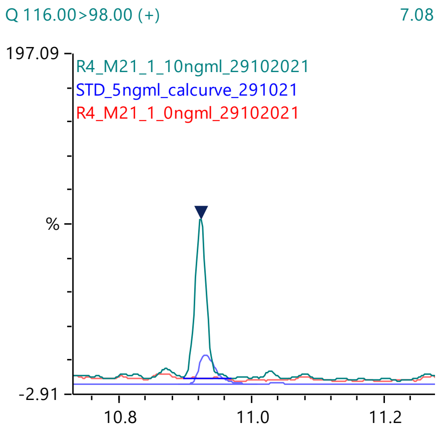

fenamidone RPA

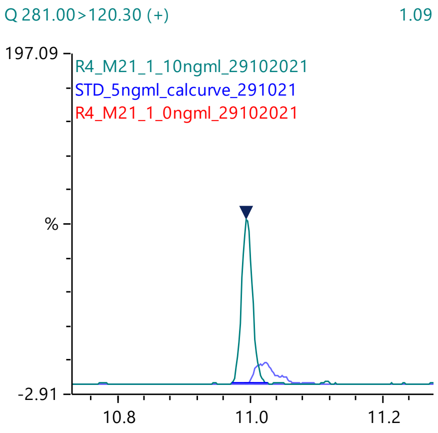

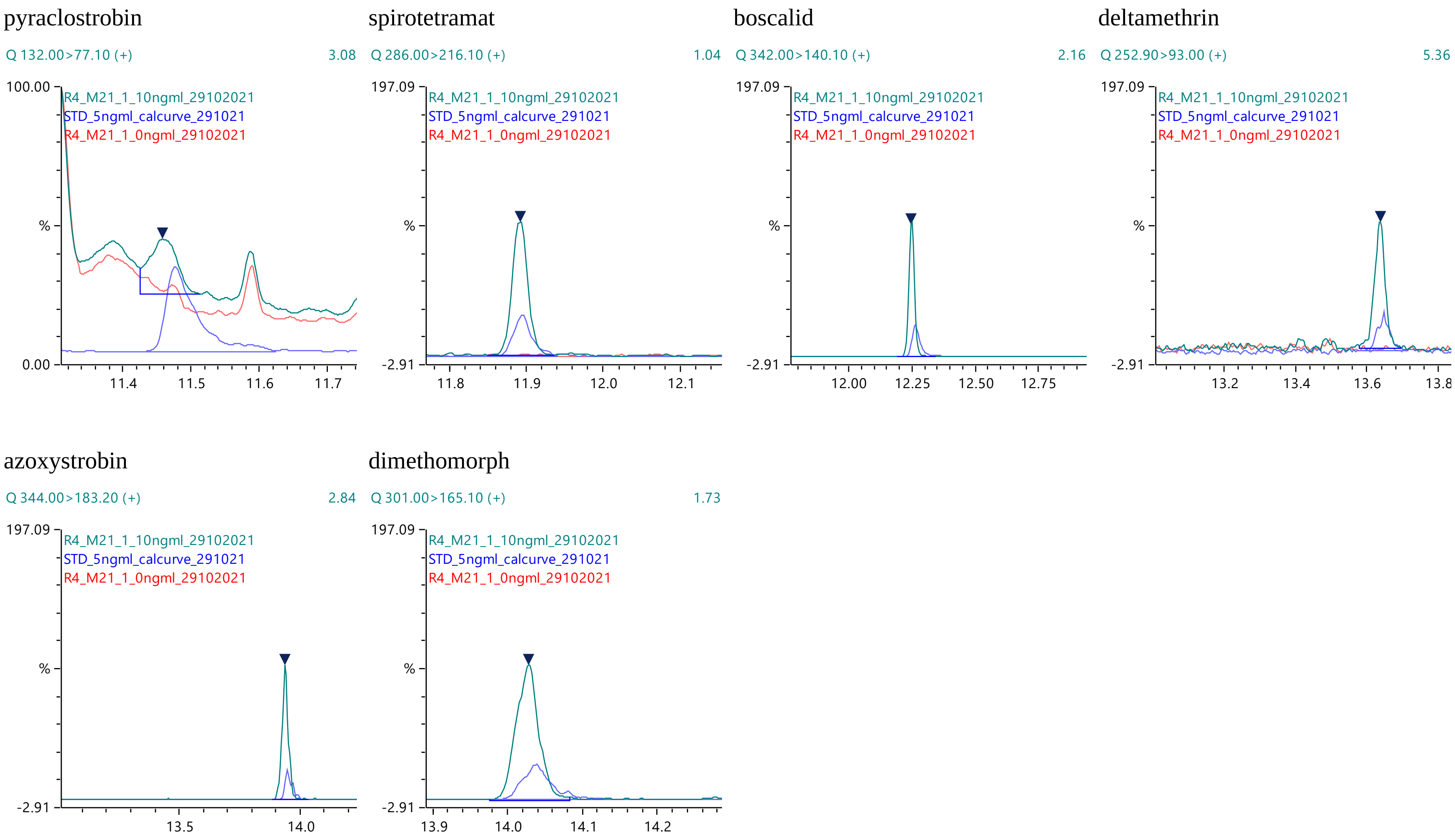

Fig S4 Chromatograms illustrating selectivity and specificity of the method. Chromatograms depict native and isotopically labelled internal standards, green (R4_M21_1_10ngml_29102021) spiked to skeletic regosol (SR) at the lowest fortification level (10 ng/g), blue (STD_5ngml_calcurve_291021) the lowest calibration point (5 ng/mL) in the matrix matched calibration curve of SR, and red (R4_M21_1_0ngml_29102021) is the blank (unspiked) SR.

atrazine desethyl-D7

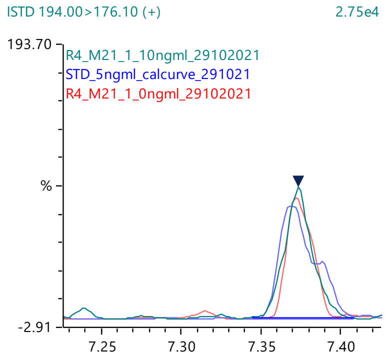

2, 6-dichlorobenzamid-D3

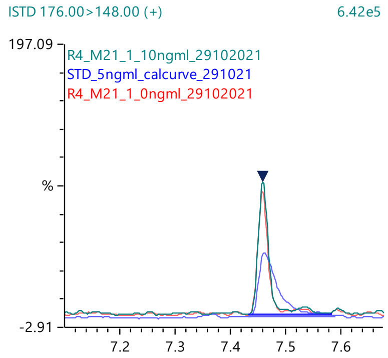

atrazine-D5

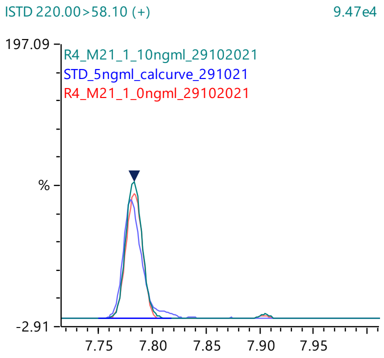

N-methyl-metribuzin-D3

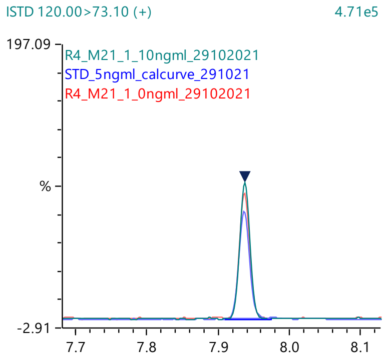

metribuzin-D3

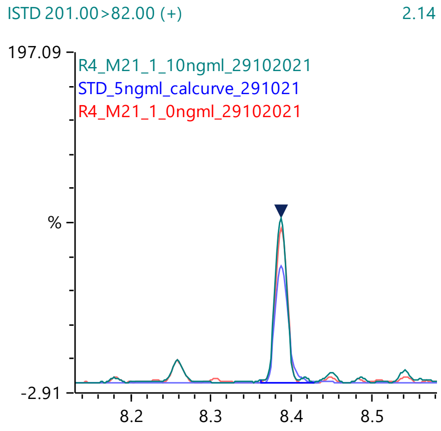

metalaxyl-D6

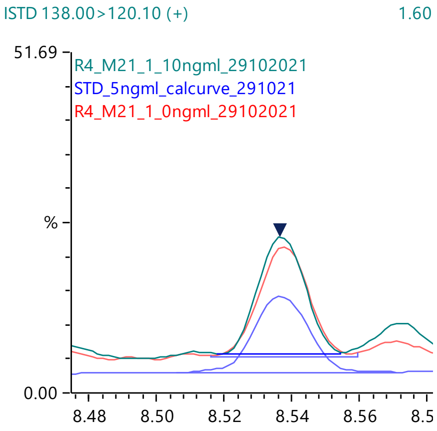

s-metolachlor-D11

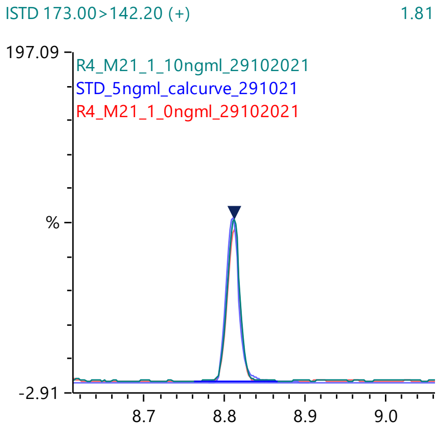

trifloxystrobin-D6

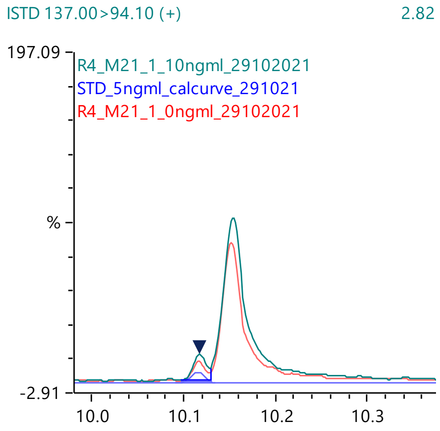

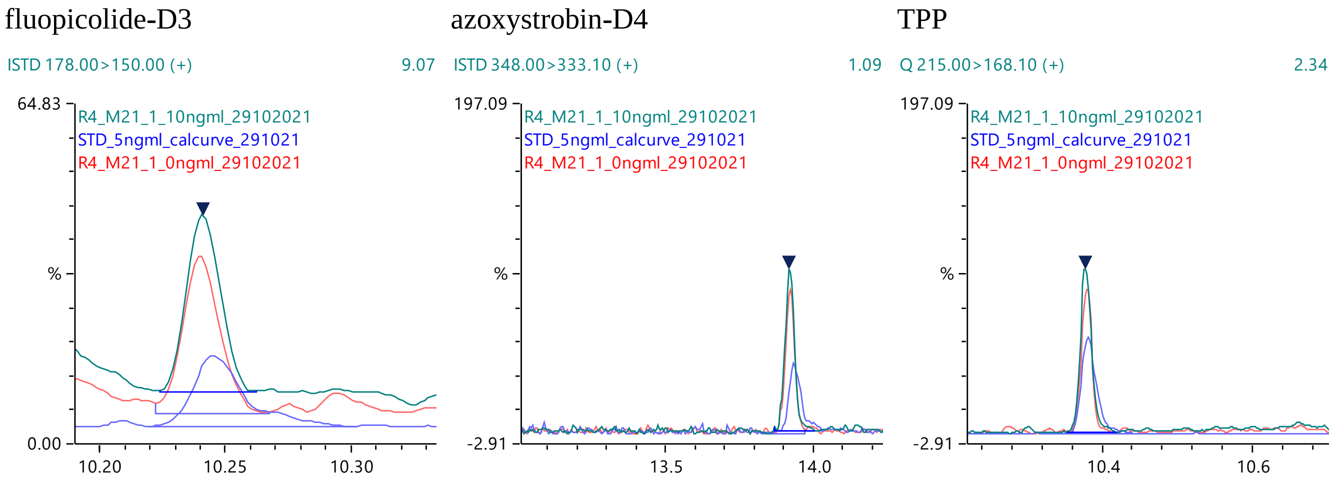

Fig S4 continued: Chromatograms of isotopically labelled internal standards (IL-IS) and syringe standard triphenyl phosphate (TPP).

4 Method validation

4.1 Slope comparison of matrix matched calibrations with the internal standard method of the soils

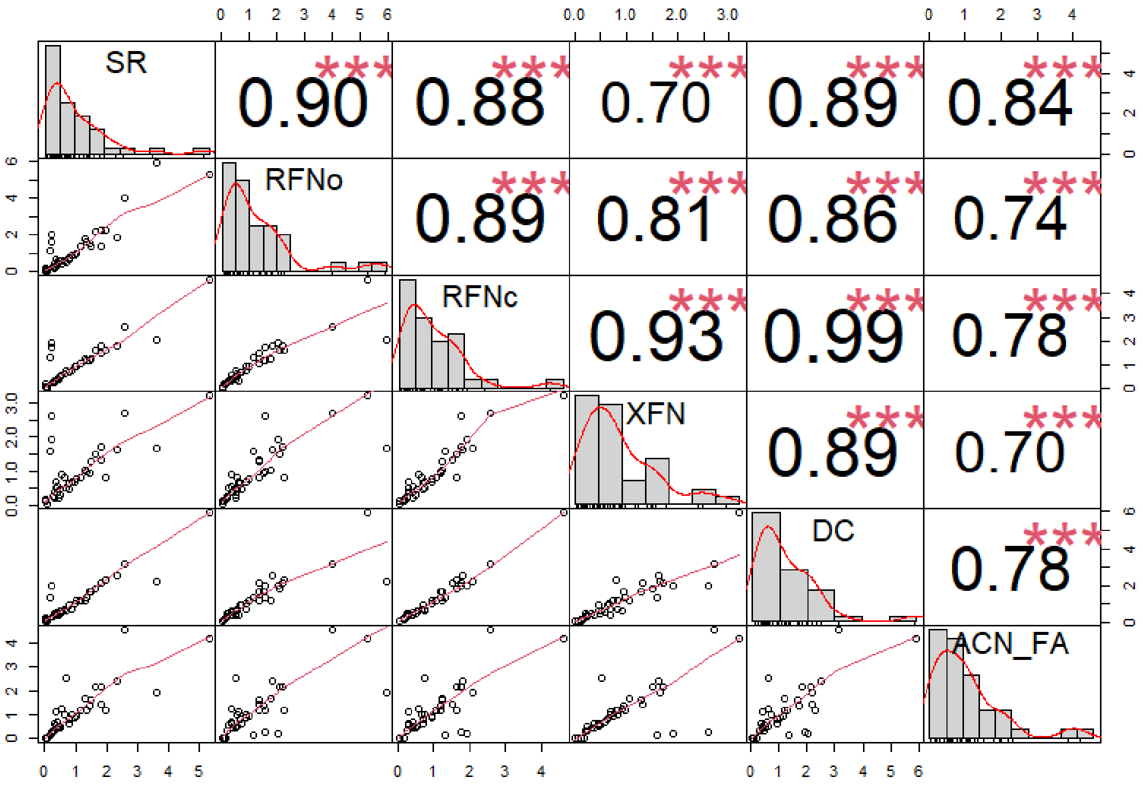

Fig S5 Scatter plot (lower half), histograms (diagonal) and correlation coefficients (r, upper half) between the slopes of 38 compounds in matrix matched calibrations calculated with the internal standard method of different soil types and acetonitrile (ACN). Histograms indicate the number of data points falling within a range or bin of values. Red lines in histograms indicate the density function. Red lines in the scatter plots are smoothed regression lines. Soil abbreviations are SR: skeletic regosol, RFNo: rhodic ferralc nitisol (organic), RFNc: rhodic ferralic nitisol (conventional), XFN: xantic ferralic nitisol, DC: dystric cambisol, ACN_FA: ACN/formic acid (2.5%, v/v). Significance code (***) is p-value <0.001.

4.2 Matrix effect in different soils

Table S5 Slope (m), coefficient of correlation (R^2) and matrix effect (ME_{ext}) calculated according to equation 1a (in the main text) of skeletic regosol. Calibration points were 5, 10, 25, 50, 75, and 100 ng/mL using the **external standard method**.

No.	compounds	Calibration in acetonitrile/formic acid (2.5%)[a]		Skeletic Regosol		
		m	R^2	m	R^2	ME_{ext} [%]
**1**	metribuzin DADK	17409	0.99	26567	1.00	53
**2**	atrazine desethyl	1985	0.94	4681	1.00	136
**3**	2,6-dichlorobenzamid	43107	0.99	68701	1.00	59
**4**	atrazine	5449	0.98	10974	1.00	101
**5**	clomazone	34151	0.99	57764	1.00	69
**6**	chlorothalonil	9203	0.99	16500	0.99	79
**7**	metribuzin DA	2179	0.98	3446	1.00	58
**8**	pirimicarb	12050	0.99	20995	1.00	74
**9**	metribuzin	9821	0.97	24898	1.00	154
**10**	ametryn	7111	0.99	16348	1.00	130
**11**	metalaxyl	16496	1.00	24180	0.99	47
**12**	prosulfocarb	4052	0.97	15916	1.00	293
**13**	dicofol	8537	0.94	80142	1.00	839
**14**	s-metolachlor	28375	0.99	53229	1.00	88
**15**	triadimenol	16284	0.99	35822	1.00	120
**16**	α-endosulfane	1183	0.97	2229	0.99	88
**17**	oxyfluorfen	1487	0.96	5367	0.99	261
**18**	fluazifop-p-butyl	16483	0.99	52845	1.00	221
**19**	β-endosulfane	3719	0.99	6216	1.00	67
**20**	trifloxystrobin CGA	86522	0.98	112338	0.93	30
**21**	cyproconazole	27036	0.99	47790	1.00	77
**22**	carfentrazone-ethyl	9210	0.96	31946	1.00	247
**23**	trifloxystrobin	39476	0.99	77019	0.99	95
**24**	benalaxyl	40787	0.96	67821	1.00	66
**25**	fluopicolide	21204	0.99	51052	1.00	141
**26**	endosulfane sulphate	9911	0.97	12754	0.99	29
**27**	tebuconazole	5568	0.99	15604	1.00	180
**28**	epoxiconazole	34628	0.99	81003	1.00	134

**29**	bifenthrin	48087	0.99	73745	0.99	53
**30**	fenamidone	10043	0.99	28644	1.00	185
**31**	benthiavalicarb-isopropyl	26353	0.99	66411	1.00	152
**32**	fenamidone RPA	3826	0.98	12811	1.00	235
**33**	pyraclostrobin	38097	0.98	23361	0.99	-39
**34**	spirotetramat	4818	0.98	11177	1.00	132
**35**	boscalid	9580	0.99	28122	1.00	194
**36**	deltamethrin	3507	0.98	10231	1.00	192
**37**	azoxystrobin	1452	0.99	5664	1.00	290
**38**	dimethomorph	9152	0.98	30073	1.00	229
	**mean**					**146**
	**min**					**-39**
	**max**					**839**
	**1st quartile**					**68**
	**3rd quartile**					**190**

[a] The two calibration curves of acetonitrile with formic acid are different because they were produced at different time points with severel weeks in between.

Table S5 continued: Slope (m), coefficient of correlation (R^2) and matrix effect (ME_{ext}) calculated according equation 1a of rhodic ferralic nitisol (RFN) organically managed (RFNo) and conventionally managed (RFNc), xanthic ferralic nitisol (XFN), and dystric cambisol (DC)). Calibration points were 5, 10, 25, 50, 75, and 100 ng/mL using the **external standard method**.

**No.**	**compounds**	**Calibration in acetonitrile/formic acid (2.5%)**		**Rhodic Ferralic Nitisol – Organically managed**			**Rhodic Ferralic Nitisol – Conventionally managed**			**Xantic Ferralic Nitisol**			**Dystric Cambisol**		
		**m**	**R^2**	**m**	**R^2**	**ME_{ext} [%]**	**m**	**R^2**	**ME_{ext} [%]**	**m**	**R^2**	**ME_{ext} [%]**	**m**	**R^2**	**ME_{ext} [%]**
**1**	metribuzin DADK	20833	0.99	21567	1.00	4	25285	0.90	21	15796	0.99	-24	28942	0.99	39
**2**	atrazine desethyl	2867	1.00	3223	1.00	12	5740	1.00	100	15822	0.98	452	6119	1.00	113
**3**	2,6-dichloro-benzamid	45759	1.00	51241	1.00	12	70831	1.00	55	64759	0.98	42	74238	1.00	62
**4**	atrazine	6162	1.00	7926	1.00	29	11725	1.00	90	12928	0.98	110	13178	1.00	114
**5**	clomazone	35317	1.00	41851	1.00	18	56987	1.00	61	53721	0.98	52	65289	1.00	85
**6**	chlorothalonil	10606	1.00	15542	0.99	47	16518	1.00	56	18720	0.98	76	18979	1.00	79
**7**	metribuzin DA	2172	1.00	2416	0.99	11	3221	1.00	48	2564	0.98	18	3523	1.00	62
**8**	pirimicarb	12801	1.00	17500	1.00	37	21585	1.00	69	19385	0.98	51	22067	1.00	72
**9**	metribuzin	12377	0.99	17802	1.00	44	27216	1.00	120	22458	0.99	81	28978	1.00	134
**10**	ametryn	9125	1.00	10975	1.00	20	18865	1.00	107	27159	0.98	198	21863	1.00	140
**11**	metalaxyl	17095	1.00	20459	1.00	20	20774	1.00	22	14911	0.98	-13	24255	0.98	42
**12**	prosulfocarb	5147	0.99	9015	0.99	75	15607	0.99	203	19355	0.98	276	19066	1.00	270
**13**	dicofol	13442	1.00	55193	0.96	311	48798	1.00	263	77251	0.98	475	80989	0.99	502
**14**	s-metolachlor	33547	1.00	45796	1.00	37	53107	1.00	58	41328	0.98	23	61760	1.00	84

**15**	triadimenol	20704	1.00	25974	1.00	25	36205	1.00	75	35452	0.98	71	43742	1.00	111
**16**	α-endosulfane	1359	1.00	1473	1.00	8	2096	1.00	54	2476	0.97	82	2601	0.99	91
**17**	oxyfluorfen	1780	0.99	2639	0.98	48	4747	1.00	167	2861	0.98	61	6144	0.99	245
**18**	fluazifop-p-butyl	21667	1.00	35969	1.00	66	49066	1.00	126	55424	0.98	156	61189	0.99	182
**19**	β-endosulfane	3842	0.99	4301	1.00	12	5700	0.99	48	3813	0.97	-1	6582	0.99	71
**20**	trifloxystrobin CGA	93640	0.98	99034	0.97	6	110972	0.93	19	112005	0.95	20	112877	0.93	21
**21**	cyproconazole	30890	1.00	34112	1.00	10	44568	1.00	44	32058	0.99	4	51601	1.00	67
**22**	carfentrazone-ethyl	11356	1.00	17111	0.98	51	28928	1.00	155	24754	0.98	118	40573	0.99	257
**23**	trifloxystrobin	49188	0.99	56226	0.99	14	75377	1.00	53	69749	0.98	42	84661	0.98	72
**24**	benalaxyl	45960	1.00	53951	1.00	17	67609	1.00	47	60409	0.98	31	73895	0.99	61
**25**	fluopicolide	31257	1.00	37127	1.00	19	51070	1.00	63	52447	0.98	68	58799	1.00	88
**26**	endosulfane sulphate	6922	1.00	18330	0.99	165	12763	1.00	84	26719	0.98	286	15713	0.99	127
**27**	tebuconazole	7276	0.99	10872	1.00	49	16818	1.00	131	22549	0.97	210	21024	0.99	189
**28**	epoxiconazole	36274	1.00	50019	0.99	38	74766	1.00	106	65870	0.98	82	90521	0.99	150
**29**	bifenthrin	54206	1.00	59444	1.00	10	68362	1.00	26	33538	0.98	-38	76397	0.98	41
**30**	fenamidone	13995	0.99	20312	1.00	45	29003	1.00	107	18607	0.98	33	35390	1.00	153
**31**	benthiavalicarb-isopropyl	34552	1.00	37688	0.99	9	63151	1.00	83	53115	0.98	54	76628	0.99	122
**32**	fenamidone RPA	5064	0.99	7490	0.99	48	12914	1.00	155	18897	0.98	273	17712	0.99	250
**33**	pyraclostrobin	31283	1.00	14691	0.99	-53	31360	1.00	0	19742	0.98	-37	25015	0.98	-20
**34**	spirotetramat	6039	0.99	5874	0.99	-3	11044	1.00	83	8793	0.98	46	17268	0.97	186
**35**	boscalid	14063	0.99	15649	1.00	11	29619	1.00	111	45536	0.98	224	39558	1.00	181

**36**	deltamethrin	4810	0.99	5099	0.99	6	9734	1.00	102	9968	0.98	107	13526	0.99	181
**37**	azoxystrobin	2409	0.99	2347	0.98	-3	5958	1.00	147	10696	0.98	344	9268	1.00	285
**38**	dimethomorph	12671	0.99	14046	1.00	11	28507	1.00	125	42664	0.98	237	43011	1.00	239
	**mean**					**34**			**89**			**113**			**136**
	**min**					**-53**			**0**			**-38**			**-20**
	**max**					**311**			**263**			**475**			**502**
	**1st quartile**					**11**			**53**			**32**			**72**
	**3rd quartile**					**45**			**118**			**187**			**182**

Table S6: Slope (m), coefficient of correlation (R^2) and matrix effect (ME_{int}) calculated according equation 1b of skeletic regosol. Calibration points were 5, 10, 25, 50, 75, and 100 ng/mL using **the internal standard method**. Rows with bold letters indicate that an isotopically labelled internal standard (IL-IS) was available. Please note that the IL-IS *n*-methyl-metribuzin-D3 was used for metribuzin desamino diketo (DADK) and desamino (DA) although it is not structure identical.

No.	compounds	Calibration in acetonitrile/formic acid (2.5%)		Skeletic Regosol		
		m	R^2	m	R^2	ME_{int} [%]
**1**	**metribuzin DADK**	**0.59**	**1.00**	**0.50**	**1.00**	**-16**
**2**	**atrazine desethyl**	**1.99**	**0.99**	**1.46**	**0.99**	**-27**
**3**	**2,6-dichlorobenzamid**	**0.53**	**0.99**	**0.60**	**0.99**	**13**
**4**	**atrazine**	**1.12**	**0.99**	**1.01**	**0.99**	**-10**
5	clomazone	6.98	0.99	5.33	0.99	-24
6	chlorothalonil	0.32	1.00	0.31	0.99	-3
**7**	**metribuzin DA**	**0.07**	**1.00**	**0.07**	**0.99**	**-11**
8	pirimicarb	0.41	1.00	0.40	1.00	-5
**9**	**metribuzin**	**1.02**	**1.00**	**0.97**	**1.00**	**-5**
10	ametryn	0.72	1.00	0.64	1.00	-12
**11**	**metalaxyl**	**0.84**	**1.00**	**0.87**	**0.99**	**4**
12	prosulfocarb	0.21	0.99	0.57	1.00	171
13	dicofol	1.56	0.99	3.62	1.00	131
**14**	**s-metolachlor**	**0.32**	**1.00**	**0.28**	**1.00**	**-12**
15	triadimenol	0.18	1.00	0.19	1.00	3
16	α-endosulfane	0.01	1.00	0.01	0.99	-10
17	oxyfluorfen	0.02	0.99	0.03	0.99	66
18	fluazifop-p-butyl	0.19	1.00	0.28	1.00	47
19	β-endosulfane	0.04	1.00	0.03	0.99	-21
20	trifloxystrobin CGA	5.62	0.99	3.47	0.96	-38
21	cyproconazole	1.75	0.99	1.50	0.99	-14
22	carfentrazone-ethyl	0.60	0.98	1.00	0.99	67
**23**	**trifloxystrobin**	**2.54**	**0.99**	**2.31**	**0.99**	**-9**
24	benalaxyl	2.67	1.00	2.06	0.99	-23
**25**	**fluopicolide**	**1.56**	**1.00**	**1.72**	**1.00**	**10**
26	endosulfane sulphate	0.71	0.99	0.43	1.00	-39
27	tebuconazole	0.40	1.00	0.52	0.99	32

28	epoxiconazole	2.51	1.00	2.73	1.00	9
29	bifenthrin	3.47	1.00	2.52	0.99	-27
30	fenamidone	0.74	1.00	0.97	1.00	31
31	benthiavalicarb-isopropyl	1.91	1.00	2.24	1.00	17
32	fenamidone RPA	0.28	1.00	0.43	1.00	55
33	pyraclostrobin	2.77	0.99	0.80	0.99	-71
34	spirotetramat	0.36	0.99	0.38	1.00	6
35	boscalid	1.74	0.99	1.29	1.00	-26
36	deltamethrin	0.64	0.99	0.47	1.00	-27
**37**	**azoxystrobin**	**0.26**	**0.99**	**0.26**	**1.00**	**2**
38	dimethomorph	1.67	0.99	1.37	1.00	-18
	**mean**	**8**			**mean**	**6**
	**min**	**50**			**min**	**-71**
	**max**				**max**	**171**
	**Structure identical IL-IS (n = 11)**					
	**Mean**					**-6**
	**min**					**-27**
	**max**					**13**
	**1st quartile**					**-11**
	**3rd quartile**					**3**
	**Non structure identical IL-IS (n = 27)**					
	**mean**					**10**
	**min**					**-71**
	**max**					**171**
	**1st quartile**					**-23**
	**3rd quartile**					**31**

Table S6 continued: Slope (m), coefficient of correlation (R^2) and matrix effect (ME_{int}) calculated according equation 1b of rhodic ferralic nitisol (RFN) organically managed (RFNo) and conventionally managed (RFNc), xanthic ferralic nitisol (XFN), and dystric cambisol (DC)). Calibration points were 5, 10, 25, 50, 75, and 100 ng/mL using **the internal standard method**. Rows with bold letters indicate an isotopically labelled internal standard (IL-IS) available. Please note that the IL-IS *n*-methyl-metribuzin-D3 was used for metribuzin desamino diketo (DADK) and desamino (DA) although it is not structure identical.

No.	Compounds	Calibration in acetonitrile/formic acid (2.5%)		Rhodic Ferralic Nitisol: Organically managed (RFNo)			Rhodic Ferralic Nitisol: Conventionally managed (RFNc)			Xantic Ferralic Nitisol (XFN)			Dystric Cambisol (DC)		
		m	R^2	m	R^2	ME_{int} [%]	m	R^2	ME_{int} [%]	m	R^2	ME_{int} [%]	m	R^2	ME_{int} [%]
**1**	**metribuzin DADK**	**0.60**	**1.00**	**0.58**	**0.99**	**-3**	**0.49**	**0.91**	**-17**	**0.31**	**1.00**	**-47**	**0.51**	**1.00**	**-12**
**2**	**atrazine desethyl**	**1.41**	**0.99**	**1.56**	**1.00**	**10**	**1.20**	**0.99**	**-15**	**0.95**	**0.98**	**-33**	**1.34**	**1.00**	**-14**
**3**	**2,6-dichloro-benzamid**	**0.49**	**0.99**	**0.58**	**0.99**	**18**	**0.53**	**0.98**	**8**	**0.47**	**0.99**	**-4**	**0.52**	**0.98**	**-11**
**4**	**Atrazine**	**1.02**	**1.00**	**1.00**	**1.00**	**-2**	**0.95**	**1.00**	**-7**	**0.75**	**0.99**	**-27**	**0.95**	**1.00**	**-5**
5	Clomazone	5.86	1.00	5.30	1.00	-10	4.60	1.00	-21	3.20	0.99	-45	4.69	1.00	-12
6	Chlorothalonil	0.30	1.00	0.42	0.99	38	0.32	1.00	5	0.37	1.00	23	0.34	0.99	-19
**7**	**metribuzin DA**	**0.06**	**1.00**	**0.07**	**0.99**	**7**	**0.06**	**1.00**	**1**	**0.05**	**1.00**	**-16**	**0.06**	**1.00**	**-5**
8	Pirimicarb	0.36	1.00	0.47	1.00	31	0.41	1.00	15	0.38	1.00	7	0.39	1.00	-17
**9**	**Metribuzin**	**1.06**	**1.00**	**0.94**	**1.00**	**-11**	**0.94**	**1.00**	**-11**	**0.67**	**0.99**	**-36**	**0.96**	**0.99**	**2**
10	Ametryn	0.78	1.00	0.60	1.00	-23	0.65	1.00	-16	0.83	0.99	7	0.67	1.00	12
**11**	**Metalaxyl**	**0.79**	**0.99**	**0.93**	**1.00**	**18**	**0.75**	**1.00**	**-4**	**0.69**	**0.99**	**-13**	**0.77**	**0.98**	**-17**
12	Prosulfocarb	0.23	0.99	0.41	1.00	74	0.57	0.99	144	0.91	0.99	289	0.60	0.99	48
13	Dicofol	1.39	1.00	5.95	0.99	328	2.08	0.99	50	1.65	0.97	19	2.25	1.00	-62
**14**	**s-metolachlor**	**1.52**	**0.99**	**2.07**	**1.00**	**36**	**1.92**	**1.00**	**26**	**1.91**	**0.99**	**25**	**1.95**	**0.99**	**-6**

15	Triadimenol	0.96	0.99	1.17	1.00	23	1.31	1.00	36	1.60	0.99	67	1.37	0.99	17
16	α-endosulfane	0.06	0.99	0.07	1.00	7	0.07	0.99	22	0.12	0.99	89	0.08	0.99	25
17	oxyfluorfen	0.08	0.99	0.11	1.00	34	0.17	1.00	102	0.13	1.00	59	0.19	0.99	69
18	fluazifop-p-butyl	1.00	0.99	1.62	1.00	62	1.76	1.00	76	2.60	0.99	160	1.93	0.99	19
19	β-endosulfane	0.17	1.00	0.19	1.00	11	0.20	1.00	16	0.18	0.99	2	0.21	0.99	7
20	trifloxystrobin CGA	4.26	0.99	4.03	0.99	-5	2.60	0.93	-39	2.69	0.97	-37	2.32	0.86	-42
21	cyproconazole	1.47	1.00	1.38	1.00	-6	1.07	1.00	-27	0.82	0.99	-44	1.00	0.99	-27
22	carfentrazone-ethyl	0.55	1.00	0.69	1.00	24	0.70	1.00	26	0.62	0.99	12	0.78	1.00	13
**23**	**trifloxystrobin**	**2.34**	**1.00**	**2.26**	**1.00**	**-3**	**1.78**	**0.99**	**-24**	**1.71**	**1.00**	**-27**	**1.61**	**0.97**	**-29**
24	benalaxyl	2.20	1.00	2.15	1.00	-2	1.63	0.99	-26	1.51	0.99	-32	1.53	0.97	-29
**25**	**fluopicolide**	**1.43**	**1.00**	**1.41**	**0.99**	**-1**	**1.23**	**0.99**	**-14**	**1.28**	**0.99**	**-11**	**1.37**	**0.98**	**-3**
26	endosulfane sulphate	0.32	1.00	0.67	0.99	113	0.31	0.99	-3	0.68	1.00	115	0.37	0.99	-45
27	tebuconazole	0.34	0.99	0.42	0.99	23	0.41	0.99	20	0.57	0.99	67	0.52	1.00	24
28	epoxiconazole	1.69	1.00	1.87	0.99	10	1.80	0.99	6	1.62	0.99	-5	2.08	0.98	11
29	bifenthrin	2.52	1.00	2.27	0.99	-10	1.64	0.99	-35	0.81	0.98	-68	1.76	0.97	-23
30	fenamidone	0.66	0.99	0.77	0.99	16	0.70	0.99	5	0.47	1.00	-29	0.86	0.99	12
31	benthiavalicarb-isopropyl	1.61	0.99	1.40	0.99	-13	1.51	0.99	-6	1.33	0.99	-18	1.89	0.98	34
32	fenamidone RPA	0.24	0.99	0.27	0.99	16	0.31	0.99	29	0.49	1.00	106	0.43	0.99	56
33	pyraclostrobin	1.42	1.00	0.54	0.99	-62	0.76	0.99	-47	0.51	0.99	-64	0.61	0.98	14
34	spirotetramat	0.28	0.99	0.22	0.99	-23	0.27	1.00	-6	0.22	0.99	-23	0.38	0.98	75
35	boscalid	1.43	1.00	1.80	0.99	26	1.25	1.00	-13	1.04	1.00	-27	1.12	0.99	-38
36	deltamethrin	0.51	1.00	0.56	0.99	9	0.41	0.99	-20	0.23	0.99	-56	0.38	0.98	-32
**37**	**azoxystrobin**	**0.25**	**1.00**	**0.26**	**0.99**	**5**	**0.25**	**1.00**	**0**	**0.24**	**0.99**	**-2**	**0.26**	**0.99**	**1**

38	dimethomorph	1.29	1.00	1.61	1.00	25	1.20	1.00	-7	0.98	1.00	-24	1.22	0.99	-25
						**RFNo**			**RFNc**			**XFN**			**DC**
	**mean**					**21**			**6**			**9**			**-1**
	**min**					**-62**			**-47**			**-68**			**-62**
	**max**					**328**			**144**			**289**			**75**
						**Structure identical IL-IS (n = 11)**									
	**mean**					**7**			**-5**			**-17**			**-9**
	**min**					**-11**			**-24**			**-47**			**-29**
	**max**					**36**			**26**			**25**			**2**
	**1st quartile**					**-2**			**-15**			**-30**			**-13**
	**3rd quartile**					**14**			**1**			**-7**			**-4**
						**Non structure identical IL-IS (n = 27)**									
	**mean**					**27**			**11**			**20**			**3**
	**min**					**-62**			**-47**			**-68**			**-62**
	**max**					**328**			**144**			**289**			**75**
	**1st quartile**					**-6**			**-18**			**-31**			**-26**
	**3rd quartile**					**28**			**24**			**63**			**22**

4.3 Figures of merit: Absolute recovery

Table S7 Mean of absolute recovery (expressed in %) obtained by isotopically labeled internal standards (IL-IS) from four replicates at three fortification levels (10, 25 and 50 ng/g) used during the validation of skeletic regosol. Standard deviation (STD) of the replicates are expressed in %. The IL-IS were spiked at 10 ng/g. Mean of three levels indicate the average of absolute recovery and standard deviation of all fortification levels.

No.	IL-IS	10 ng/g		25 ng/g		50 ng/g		Mean of three levels	
		Absolute Recovery	STD	Absolute Recovery	STD	Absolute Recovery	STD	Absolute recovery	STD
**1**	atrazine desethyl-D7	92	20	113	27	115	37	107	28
**2**	2,6-dichlorobenzamid-D3	79	7	84	9	93	17	86	11
**3**	atrazine-D5	93	5	93	11	93	22	93	13
**4**	*n*-methyl-metribuzin-D3	42	4	43	3	42	5	43	4
**5**	metribuzin-D3	95	6	98	15	99	16	97	12
**6**	metalaxyl-D6	90	11	86	6	81	11	86	10
**7**	s-metolachlor-D11	94	9	94	9	93	16	94	12
**8**	trifloxystrobin-D6	98	12	100	10	96	12	98	11
**9**	fluopicolide-D3	89	11	93	7	102	14	94	11
**10**	azoxystrobin-D4	117	16	125	25	127	27	123	23

5 Application of the method to real samples

5.1 Quality control and quality assurance (QA/QC)

Table S8 Absolute recoveries of two series of samples analyzed (a serie contained real soil samples n=15, rhodic ferralic nitisol organically managed without spiking n=3, sand without spiking n=3, and RFNo spiked n=3). Absolute recovery represents the mean of the values in each serie of samples and standard deviation (STD) expressed in %. Mean of two series indicates the average of absolute recovery and standard deviation of two series, and are expressed in %.

No.	IL-IS	Absolute recovery (STD) in %		Mean of two series
		Serie 1	Serie 2	Absolute recovery (STD) in %
**1**	atrazine desethyl-D7	94 (17)	98 (14)	96 (3)
**2**	2,6-dichlorobenzamid-D3	87 (8)	85 (6)	86 (1)
**3**	atrazine-D5	97 (9)	106 (9)	101 (7)
**4**	*n*-methyl-metribuzin-D3	109 (8)	107 (8)	108 (1)
**5**	metribuzin-D3	110 (16)	102 (10)	106 (5)
**6**	metalaxyl-D6	90 (8)	92 (7)	91 (1)
**7**	s-metolachlor-D11	92 (6)	102 (9)	97 (7)
**8**	trifloxystrobin-D6	104 (11)	109 (15)	107 (3)
**9**	fluopicolide-D3	111 (11)	118 (13)	114 (5)
**10**	azoxystrobin-D4	110 (11)	115 (19)	112 (4)

Table S9 Mean of relative recovery at different fortification levels (10, 25, 50 ng/g, n=2 for each level) in rhodic ferralic nitisol organically managed and limit of quantification (LOQ). Recovery means, standard deviation (STD) and relative standard deviation for reproducibility (RSD_R) are expressed in %.

No.	Compounds	Relative recovery (STD) in %			RSD_R in %			LOQ (ng/g)
		10 ng/g	25 ng/g	50 ng/g	10 ng/g	25 ng/g	50 ng/g	
**1**	metribuzin DADK	13 (3)	85 (28)	82 (7)	23	33	8	5
**2**	atrazine desethyl	99 (8)	112 (17)	107 (1)	8	15	1	1
**3**	2,6-dichlorobenzamid	105 (3)	118 (3)	114 (4)	3	3	3	0.5
**4**	atrazine	106 (17)	113 (4)	102 (3)	16	3	3	0.1
**5**	clomazone	106 (11)	113 (7)	100 (0)	11	6	0	0.25
**6**	chlorothalonil	88 (2)	79 (3)	63 (4)	2	4	6	0.1
**7**	metribuzin DA	91 (11)	99 (3)	87 (15)	13	3	17	25
**8**	pirimicarb	84 (2)	97 (4)	77 (6)	2	4	8	5
**9**	metribuzin	94 (10)	113 (11)	99 (4)	11	9	4	0.5
**10**	ametryn	99 (11)	105 (5)	87 (5)	11	5	6	1
**11**	metalaxyl	120 (1)	115 (17)	112 (2)	1	15	2	10
**12**	prosulfocarb	79 (2)	121 (11)	107 (10)	2	9	9	0.1
**13**	s-metolachlor	118 (0)	114 (22)	106 (1)	0	19	1	1
**14**	dicofol	120 (0)	94 (20)	129 (1)	0	22	1	0.1
**15**	trifloxystrobin CGA	200 (6)	113 (26)	117 (9)	3	23	8	1
**16**	triadimenol	115 (1)	111 (19)	104 (0)	1	17	0	5
**17**	α-endosulfane	103 (16)	104 (22)	100 (1)	15	21	1	1
**18**	oxyfluorfen	87 (16)	123 (42)	122 (5)	18	34	4	5
**19**	fluazifop-p-butyl	69 (0)	80 (2)	83 (0)	0	3	0	0.1
**20**	cyproconazole	123 (3)	113 (16)	92 (8)	2	14	9	5
**21**	β-endosulfane	102 (5)	108 (19)	102 (2)	5	17	2	0.5
**22**	carfentrazone-ethyl	15 (2)	60 (0)	60 (4)	11	0	7	0.5
**23**	trifloxystrobin	109 (1)	117 (9)	99 (9)	1	7	9	5
**24**	benalaxyl	185 (3)	107 (11)	96 (12)	2	10	12	0.1
**25**	fluopicolide	93 (1)	109 (14)	102 (4)	1	12	4	5
**26**	endosulfane sulphate	77 (5)	94 (27)	98 (7)	7	29	8	0.1
**27**	tebuconazole	74 (0)	114 (14)	104 (4)	0	12	3	0.1
**28**	epoxiconazole	68 (7)	107 (14)	111 (2)	10	14	2	0.1
**29**	bifenthrin	93 (4)	103 (10)	103 (0)	4	9	0	5
**30**	fenamidone	50 (1)	53 (8)	50 (3)	3	14	6	5

**31**	benthiavalicarb-isopropyl	71 (8)	101 (3)	111 (1)	11	3	1	0.5
**32**	fenamidone RPA	28 (1)	72 (1)	67 (5)	3	1	8	0.1
**33**	pyraclostrobin	92 (4)	95 (3)	103 (1)	4	3	1	10
**34**	spirotetramat	31 (14)	75 (2)	62 (2)	46	3	3	1
**35**	boscalid	111 (2)	103 (2)	96 (4)	2	2	5	0.1
**36**	deltamethrin	98 (8)	101 (9)	104 (6)	8	9	6	5
**37**	azoxystrobin	85 (12)	111 (4)	97 (6)	14	4	7	0.1
**38**	dimethomorph	93 (11)	115 (0)	107 (8)	12	0	7	1
	Mean							2.8
	Min							0.1
	Max							25

Table S10: Mean blank concentrations (Conc.) of unspiked skeletic regosol (n=12 in total of four replicates/day), sand sample (n=6 in total of triplicates/day), and rhodic ferralic nitisol organically managed (n=6 in total of triplicates/day). Standard deviation (STD) and coefficient of variation (CV) are expressed in %. Empty cells indicate no performance at this day.

**metribuzin desamino diketo**	**Skeletic regosol LOQ[a] 10 ng/g[b]**			**Sand sample**			**Rhodic ferralic nitisol organic, LOQ 5 ng/g[c]**		
	**Conc. [ng/g]**	**STD [%]**	**CV [%]**	**Conc. [ng/g]**	**STD [%]**	**CV [%]**	**Conc. [ng/g]**	**STD [%]**	**CV [%]**
**1st day**	6.07	0.34	5.62	7.7	1.6	20.1	10.5	1.4	13.6
**2nd day**	4.73	0.20	4.18	6.4	1.6	25.0	9.9	1.3	13.0
**3rd day**	2.76	0.69	25.00						
**Mean of days**	4.52	0.41	11.60	7.1	1.6	22.5	10.2	1.4	13.3

[a] LOQ: limit of quantification; [b] Table 2; [c] Table S8

6 Supplementary References

Acosta-Dacal A, Rial-Berriel C, Díaz-Díaz R, del Mar Bernal-Suárez M, Luzardo OP (2021): Optimization and validation of a QuEChERS-based method for the simultaneous environmental monitoring of 218 pesticide residues in clay loam soil. Sci. Total Environ. 753, 142015. https://doi.org/10.1016/j.scitotenv.2020.142015

Anastassiades M, Maštovská K, Lehotay SJ (2003): Evaluation of analyte protectants to improve gas chromatographic analysis of pesticides. J. Chromatogr. A 1015, 163-184. https://doi.org/10.1016/S0021-9673(03)01208-1

MINAG (2008): Lista Oficial de Plaguicidas Autorizados. Registro Central de Plaguicidas. República de Cuba

Pérez-Consuegra N, Montano-Pérez M (2021): Los Plaguicidas Altamente Peligrosos en Cuba, IPEN/ACTAF/RAPAL. Editora Agroecológica 56.

PPDB (2023): Pesticide properties database, agriculture & environment research unit (AERU), University of Hertfordshire, UK, https://sitem.herts.ac.uk/aeru/ppdb/en/atoz.htm, accessed 22-03-2023

v
